# Dual inhibition of xCT and GGCT induces ferroptosis in glioblastoma cells by depleting cysteine and disrupting redox homeostasis

**DOI:** 10.1038/s41420-026-03108-9

**Published:** 2026-04-15

**Authors:** Masaya Mori, Hiromi Ii, Mai Matsumura, Yuhi Sone, Haruna Kumamoto, Kana Sakurai, Teruna Fujino, Nanami Nihei, Nana Hongo, Kozue Nose, Takahiro Matsumoto, Mitsugu Fujita, Susumu Nakata

**Affiliations:** 1 Laboratory of Clinical Oncology, Kyoto Pharmaceutical University, Kyoto, Japan; 2 Laboratory of Public Health, Kyoto Pharmaceutical University, Kyoto, Japan; 3 Center for Medical Education and Clinical Training, Kindai University Faculty of Medicine, Osaka-Sayama, Japan

**Keywords:** CNS cancer, CNS cancer

## Abstract

Glioblastoma is the most aggressive and treatment-resistant brain tumor. Ferroptosis, an iron-dependent form of regulated cell death caused by lipid peroxidation, has emerged as a promising therapeutic strategy; however, intrinsic resistance to ferroptosis limits its therapeutic efficacy. Here, we demonstrate that metabolic depletion of cysteine through dual inhibition of exogenous and endogenous sources represents a novel approach to overcome this resistance. While inhibition of xCT suppresses cystine uptake and induces ferroptosis, we identified γ-glutamylcyclotransferase (GGCT), a key enzyme in glutathione (GSH) degradation, as a metabolic compensation pathway that regenerates cysteine to sustain redox homeostasis. Blocking both xCT and GGCT synergistically depleted intracellular cysteine and GSH, leading to excessive accumulation of reactive oxygen species (ROS), lipid peroxidation, and ferroptotic cell death in glioblastoma cells. Importantly, dual inhibition markedly suppressed tumor growth in vivo and enhanced oxidative stress in tumor tissues, as evidenced by 4-hydroxynonenal accumulation. These findings uncover a previously unrecognized mechanism by which GGCT confers ferroptosis resistance by maintaining intracellular redox balance. Targeting the xCT–GGCT axis effectively disrupts redox homeostasis and eliminates metabolic plasticity that underlies ferroptosis resistance in glioblastoma. This study provides a mechanistic and translational rationale for developing dual inhibition of xCT and GGCT as a promising therapeutic strategy against this lethal and therapy-refractory cancer.

## Introduction

Glioblastoma (GBM), the most aggressive malignant brain tumor among adult-onset central nervous system malignancies, is characterized by its highly invasive nature [1]. GBM exhibits rapid cellular proliferation and extensive infiltration into surrounding normal brain tissue, making complete surgical resection and effective radiotherapy difficult [2]. Furthermore, resistance to chemotherapeutic agents, including the standard treatment temozolomide, is common. Despite the adoption of multimodal therapeutic strategies, the median overall survival remains approximately 14.6 months, and the 5-year survival rate is less than 10% [3]. The resistance of GBM to treatment is attributed to genetic and phenotypic heterogeneity of the tumor cells, as well as the presence of glioblastoma stem cells (GSC) within the tumor [4, 5]. GSC possess self-renewal capacity and multipotency, exhibit high resistance to chemotherapy and radiotherapy, and are major contributors to tumor recurrence [6]. Therefore, development of novel therapeutic strategies against GBM is an urgent issue; with this in mind, recent research has focused on targeting the metabolic properties of tumors [7].

System xc⁻ is an anionic amino acid antiporter that imports one molecule of extracellular cystine in exchange for one molecule of intracellular glutamate. The xCT subunit, which is encoded by the *SLC7A11* gene, is responsible for transporter activity and forms a heterodimer with the heavy chain 4F2hc, which is required for membrane localization. xCT plays a crucial role in resistance of cancer cells to oxidative stress by functioning as the active subunit of the system xc⁻ transporter [8–10]. System xc⁻ imports cystine, which is reduced intracellularly to cysteine, which serves as a precursor for glutathione (GSH) synthesis. GSH is the principal intracellular antioxidant that protects cells from oxidative stress by scavenging reactive oxygen species (ROS). Overexpression of xCT is essential for the survival of rapidly proliferating cancer cells and cancer stem cells, which are highly susceptible to oxidative stress [11–13]. Inhibition of xCT blocks the supply of cysteine, resulting in depletion of intracellular GSH and disruption of oxidative stress defense mechanisms, thereby inducing oxidative stress-dependent cell death mechanisms such as ferroptosis. Accordingly, therapeutic strategies targeting system xc⁻, and its interaction with CD44 variants, have emerged as promising approaches to targeting GSC [14, 15].

Erastin, a representative xCT inhibitor, binds to xCT directly, thereby inhibiting cystine uptake and lowering intracellular GSH levels via downstream pathways. It is classified as a Class I Ferroptosis Inducer that induces GPX4-independent ferroptosis [8, 13, 16]. Ferroptosis is a form of iron-dependent cell death characterized by lipid peroxidation and membrane damage. During this process, free radicals oxidize the polyunsaturated fatty acids in the cell membrane; this process is catalyzed by iron ions, which thereby amplify oxidative stress. This leads to accumulation of lipid peroxides via the Fenton reaction, ultimately resulting in membrane disruption. Since the supply of cysteine is crucial for preventing ferroptosis, restricting cysteine metabolism significantly impairs the oxidative stress defense mechanisms of tumor cells, and increases their susceptibility to cell death [8]. Consequently, targeting xCT and cysteine-mediated metabolic pathways is as a promising therapeutic strategy for inducing ferroptosis in cancer cells.

Recent studies report high levels of γ-glutamylcyclotransferase (GGCT) expression by various cancers, including GBM [17]. GGCT is an enzyme involved in the γ-glutamyl cycle, playing a key role in both the synthesis and degradation of reduced GSH. In cancer cells, GGCT functions as a major source of intracellular cysteine by degrading γ-glutamylcysteine [18]. Indeed, knockout of GGCT reduces cysteine levels in fibroblasts [19]. It was shown that GGCT cleaves γ-glutamylcysteine to generate cysteine [20], suggesting that it may serve as an endogenous cysteine source independent of system xc⁻. However, its contribution to cysteine homeostasis in GBM remains unclear. Downregulation of GGCT exerts antitumor effects by increasing intracellular ROS levels; this supports the idea that GGCT plays a central role in maintaining intracellular antioxidant homeostasis and resistance to cell death [21–23]. Our research group has independently developed a GGCT inhibitor called pro-GA, and reported its antitumor efficacy in models of prostate and breast cancer [23, 24]; however, to date, no studies have examined application of pro-GA in the context of GBM.

Here, we hypothesize that GGCT, a potential endogenous source of cysteine other than xCT-mediated cystine uptake, may contribute to resistance to therapies involving xCT inhibition, such as erastin. Therefore, we asked whether inhibiting GGCT would enhance the ferroptosis-inducing effects of xCT inhibition in GBM by blocking the supply of intracellular cysteine. Furthermore, we evaluated the therapeutic efficacy of combining xCT and GGCT inhibitors in a mouse model of glioblastoma, and obtained evidence that this combination induces ferroptosis.

## Results

### GGCT and xCT are highly expressed in glioblastoma cells, and their combined inhibition exerts synergistic antiproliferative effects

**Fig. 1:**
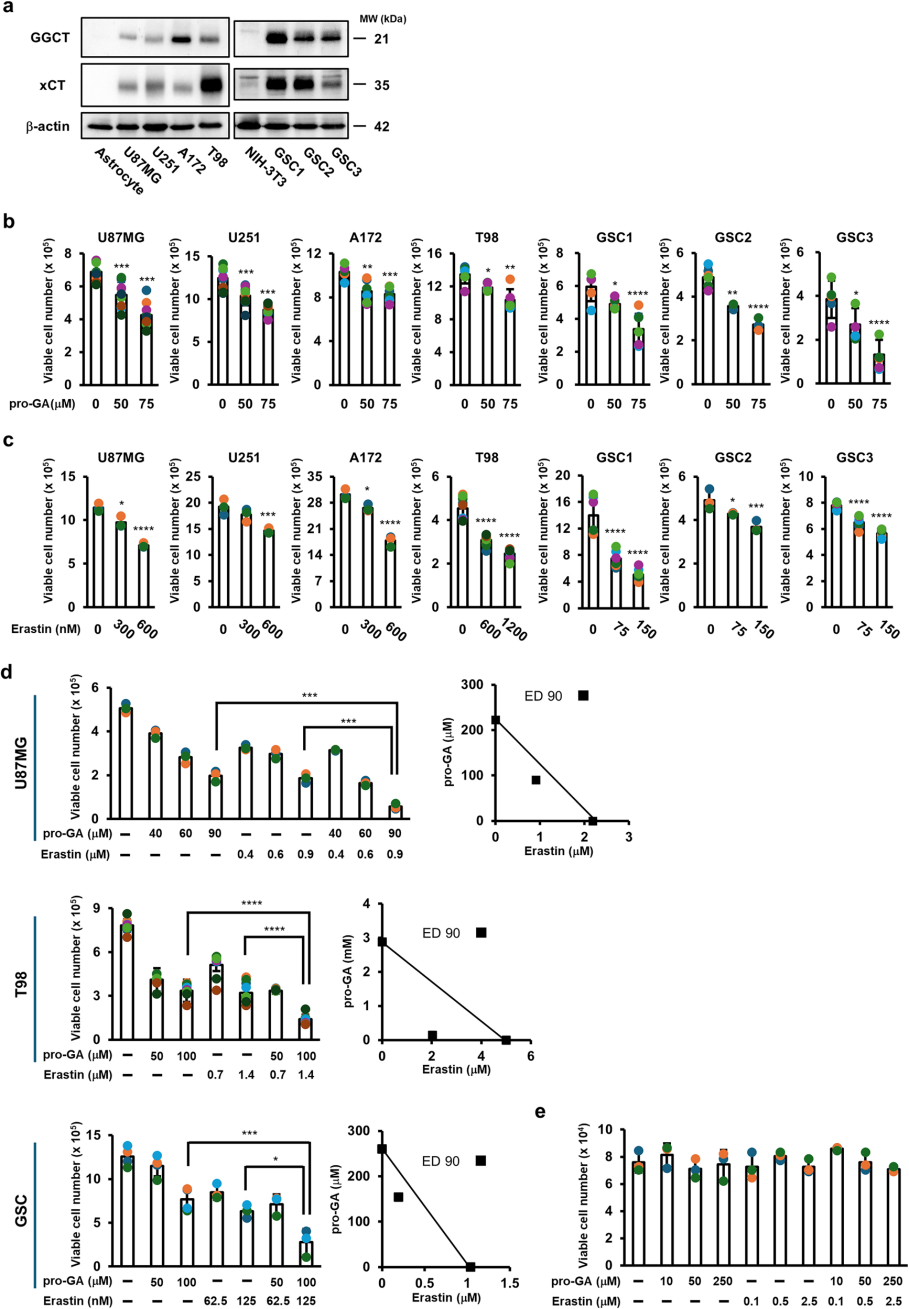
Pro-GA and erastin act synergistically to inhibit proliferation of glioblastoma cells. **a** Levels of GGCT and xCT expression in astrocytes, U87MG, U251, A172, T98, NIH-3T3, and three different GSC lines were compared by western blotting. Glioblastoma cells were treated for 3 days with pro-GA (50 or 75 μM; **b**) or erastin (75, 150, 300, 600, or 1,200 nM; **c**), and viable cell numbers were quantified ((b) U87MG, U251: n = 9; A172, T98, GSC1, GSC3: n = 6; GSC2: 0 µM: n = 6, 50 and 100 µM: n = 3; **c** T98: n = 9; U251, GSC1, GSC3: n = 6; U87MG, A172, GSC2: n = 3). **d** U87MG, T98, or GSC1 cells were treated for 3 days with pro-GA and/or erastin at the indicated concentrations, and viable cells were counted. Isobolograms at the ED90 level are shown (U87MG: n = 3, T98: n = 9, GSC: DMSO, pro-GA 50 or 100 μM; n = 4, erastin 0.0625 or 0.125 μM, pro-GA 50 μM with erastin 0.0625 μM, pro-GA 50 μM with erastin 0.125 μM; n = 3). **e** Human peripheral blood mononuclear cells (hPBMCs) were treated for 3 days with pro-GA or erastin at the indicated concentrations, and viable cells were counted (n = 3). Data are presented as mean ± SD. Statistical analysis was performed using ANOVA with Bonferroni’s multiple comparison test. *p < 0.05, **p < 0.01, ***p < 0.001, ****p < 0.0001.

We first examined the expression of GGCT and xCT in glioblastoma cells by western blot analysis. Compared with normal astrocytes, all human glioblastoma cell lines analyzed (U87MG, U251, A172, and T98) showed markedly elevated expression of both GGCT and xCT. Similarly, all three GSC lines (GSC1–3) exhibited higher expression levels of these proteins compared with non-cancerous NIH-3T3 cells (Fig. 1a).

We next assessed the antiproliferative effects of the GGCT inhibitor pro-GA and the xCT inhibitor erastin. Pro-GA significantly suppressed proliferation of all glioblastoma cell lines and GSCs in a dose-dependent manner (Fig. 1b). Likewise, erastin suppressed proliferation of all tested cell lines and GSCs in a dose-dependent manner (Fig. 1c).

To determine whether combined inhibition of GGCT and xCT results in synergistic effects, we treated cells with pro-GA and erastin at a fixed ratio for 3 days. Combination index (CI) analysis revealed statistically significant synergism in U87MG, T98, and GSC1 cells, with CI values of 0.781, 0.456, and 0.775 at the ED90 level, respectively (Fig. 1d). These findings demonstrate that dual inhibition of GGCT and xCT synergistically suppresses proliferation of glioblastoma cells (Fig. 1e). Importantly, even at high concentrations, single or combined treatment for 3 days did not affect the viability of human peripheral blood mononuclear cells (hPBMCs), indicating that the inhibitory effects were specific to tumor cells.

### The combination of pro-GA and erastin induces ferroptosis in glioblastoma cells

**Fig. 2:**
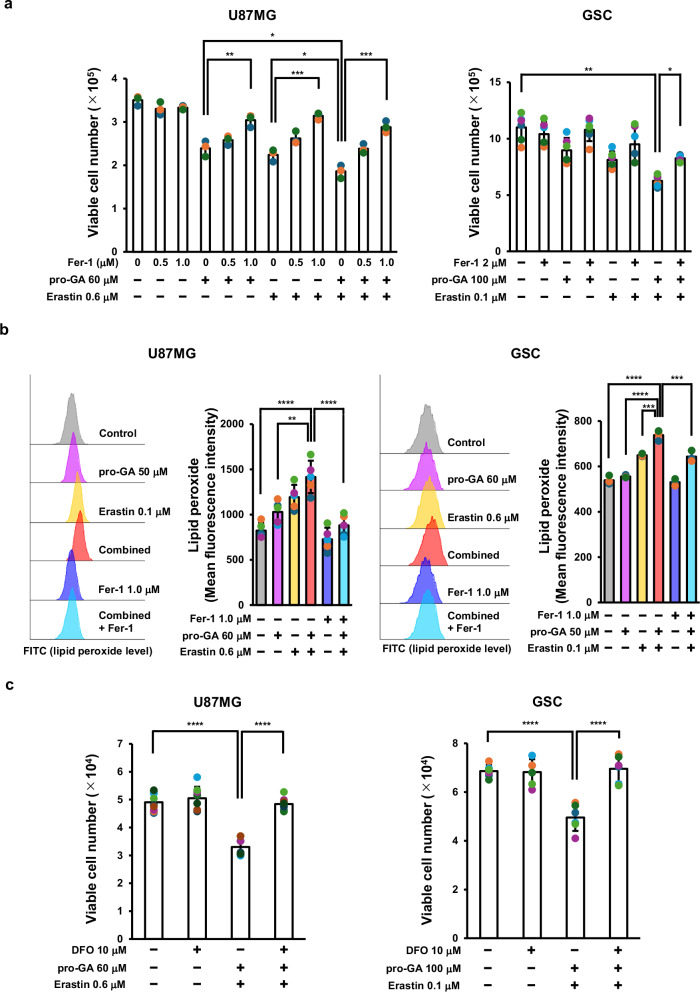
Combined treatment with pro-GA and erastin results in antiproliferative activity via induction of ferroptosis. **a** U87MG cells were treated for 3 days with pro-GA (60 μM), erastin (0.6 μM), or a combination of both, with or without Ferrostatin-1 (Fer-1; 0.5 or 1 μM). GSC were similarly treated with pro-GA (100 μM), erastin (0.1 μM), or the combination, with or without Fer-1 (2 μM). Viable cell numbers were determined (U87MG, GSC: n = 6). **b** Lipid peroxidation was analyzed by flow cytometry using BODIPY 581/591 C11. U87MG cells were treated for 3 days with pro-GA (60 μM), erastin (0.6 μM), or the combination, with or without Fer-1 (1 μM). GSC were treated under the same conditions with pro-GA (100 μM), erastin (0.1 μM), and/or Fer-1 (2 μM). Representative histograms of lipid peroxidation and mean fluorescence intensity (MFI) values are shown (U87MG, GSC: n = 3). **c** The iron chelator deferoxamine (DFO) reversed the antiproliferative effect of the pro-GA plus erastin combination. U87MG cells were treated for 3 days with pro-GA (60 μM) and erastin (0.6 μM), with or without DFO (10 μM). GSC were treated similarly with pro-GA (100 μM) and erastin (0.1 μM), with or without DFO (10 μM), and viable cell numbers were counted (U87MG, GSC: n = 6). Data are presented as mean ± SD. Statistical analysis was performed using ANOVA with Bonferroni’s multiple comparison test. *p < 0.05, **p < 0.01, ***p < 0.001, ****p < 0.0001.

To elucidate the mechanism underlying the antiproliferative effects of the pro-GA and erastin combination, we evaluated the impact of the ferroptosis-specific inhibitor Ferrostatin-1 (Fer-1) on U87MG cells and GSC subjected to combination therapy. In both cell types, the growth inhibition induced by the combination treatment was reversed significantly by Fer-1 (Fig. 2a). Phase-contrast microscopy revealed a reduction in the density of U87MG cells, as well as a reduction in the size of GSC spheroids. Upon combination treatment, both of which were suppressed by Fer-1 (Fig. S1a). These results suggest that the combination treatment inhibits growth by inducing ferroptosis.

Next, we evaluated accumulation of intracellular lipid peroxides, a hallmark of ferroptosis, using flow cytometry. Combined treatment increased lipid peroxide levels significantly in both U87MG cells and GSC, a phenomenon suppressed by Fer-1, indicating that combined treatment causes ferroptosis-dependent accumulation of lipid peroxides (Fig. 2b).

As ferroptosis is an iron-dependent form of cell death, we next examined the impact of the iron chelator deferoxamine (DFO). DFO reversed the growth inhibition induced by combined treatment significantly (Fig. 2c). Collectively, these findings indicate that the combination of pro-GA and erastin exerts a synergistic antiproliferative effect on glioblastoma cells via ferroptosis, which is characterized by lipid peroxidation and iron dependency.

### Dual inhibition of GGCT and xCT induces ferroptosis through cysteine depletion and redox collapse

**Fig. 3:**
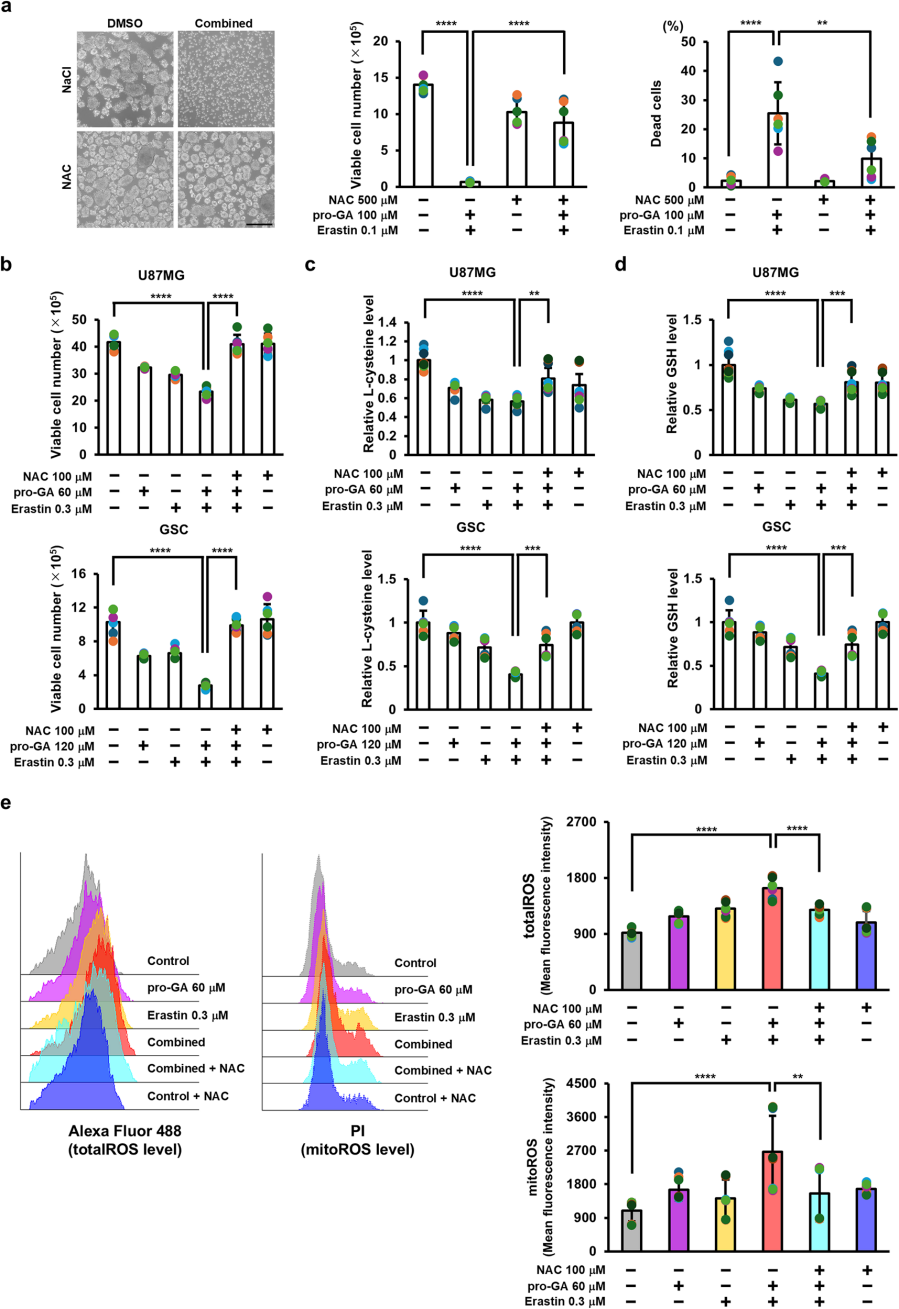
Depletion of intracellular L-cysteine by combined treatment with pro-GA and erastin induces ferroptosis in glioblastoma cells. **a** *N*-acetylcysteine (NAC) rescues ferroptotic cell death in GSC1 cells induced by combined treatment with pro-GA and erastin. GSC1 cells were treated for 4 days with pro-GA (100 μM), erastin (100 nM), or the combination, in the presence or absence of NAC (500 μM). Representative spheroid images, viable cell counts, and cell death ratios are shown. Combined treatment markedly impaired spheroid formation, reduced cell viability, and increased cell death, all of which were rescued by NAC. Scale bar, 100 μm (n = 6). **b** U87MG cells were treated with pro-GA (60 μM), erastin (0.3 μM), or both, with or without NAC (100 μM), and viable cells were counted. GSC were treated similarly with pro-GA (120 μM), erastin (0.3 μM), or the combination, with or without NAC (100 μM), and viable cells were counted (U87MG, GSC: n = 6). Intracellular L-cysteine (**c**) and reduced GSH (**d**) levels in U87MG cells and GSC treated with pro-GA, erastin, or the combination, with or without NAC, were measured by LC–MS/MS. Values were normalized to the control group. Combined treatment significantly reduced intracellular L-cysteine and GSH levels in both U87MG cells and GSC, and these reductions were restored by NAC (U87MG: control, pro-GA, erastin, combination, n = 6; NAC, n = 9; GSC, n = 6). **e** U87MG cells were treated for 3 days with pro-GA (60 μM), erastin (0.3 μM), or both, with or without NAC (100 μM). Representative histograms and mean fluorescence intensity (MFI) values of total and mitochondrial reactive oxygen species (ROS) are shown (total ROS: n = 9; mitochondrial ROS: control, single treatments, combination, n = 9; NAC and combination + NAC, n = 6). Data are presented as mean ± SD. Statistical analysis was performed using ANOVA with Bonferroni’s multiple comparison test. *p < 0.05, **p < 0.01, ***p < 0.001, ****p < 0.0001.

To investigate the mechanism by which the combination of pro-GA and erastin induces ferroptosis, we assessed whether the mechanism involved disrupting intracellular redox homeostasis. After 4 days, combined treatment of GSC1 cells led to a marked reduction in spheroid formation and a significant reduction in viable cell numbers, while at the same time increasing the cell death ratio. These effects were rescued by addition of *N*-acetylcysteine (NAC), which restored spheroid morphology, cell viability, and the death ratio to levels comparable with those in the control group (Fig. 3a). A higher concentration of NAC was used in the spheroid assay because rescue was assessed at day 4, when oxidative stress and cell death were more pronounced. These results indicate that combined treatment induces oxidative stress-dependent ferroptotic cell death in GSC.

Next, we analyzed metabolic changes in U87MG cells and GSC after 3 days of treatment, i.e., prior to the full onset of cell death. Although both pro-GA and erastin inhibited proliferation when used alone, combined treatment led to a significantly greater reduction in cell viability, which was reversed completely by NAC (Fig. 3b).

High-performance liquid chromatography analysis revealed that combined treatment reduced intracellular cysteine levels significantly; these levels were restored by NAC supplementation (Fig. 3c). Similarly, levels of reduced GSH decreased upon combined treatment, and then recovered after addition of NAC (Fig. 3 d). Given that GSH is essential for removal of lipid peroxides, its depletion reflects metabolic dysfunction due to cysteine starvation. These findings are consistent with those of previous studies showing that cysteine is the rate-limiting precursor for GSH synthesis [25].

In parallel with the decrease in cysteine and GSH levels in GSC, we observed a significant increase in both total ROS and mitochondrial ROS. Importantly, this ROS accumulation was effectively eliminated by NAC treatment, further confirming that combined inhibition induces oxidative stress through cysteine depletion (Fig. 3e).

To further clarify the terminal execution mechanism of ferroptosis induced by dual inhibition, we examined whether the ACSL4/LPCAT3 pathway or GPX4 suppression was affected. Western blot analysis showed that protein expression levels of ACSL4 and LPCAT3 were not significantly altered by the combination treatment (Fig. S2a). Similarly, GPX4 protein expression remained unchanged (Fig. S2a). In addition, total GPX enzymatic activity measured in the presence of abundant exogenous GSH showed no significant difference between groups (Fig. S2b), indicating that the intrinsic catalytic activity of GPX4 was not directly suppressed. These findings suggest that enhanced lipid peroxidation was not driven by induction of the ACSL4/LPCAT3 or by downregulation of GPX4 expression or intrinsic activity, but rather that the depletion of GSH was the primary cause.

**Fig. 4:**
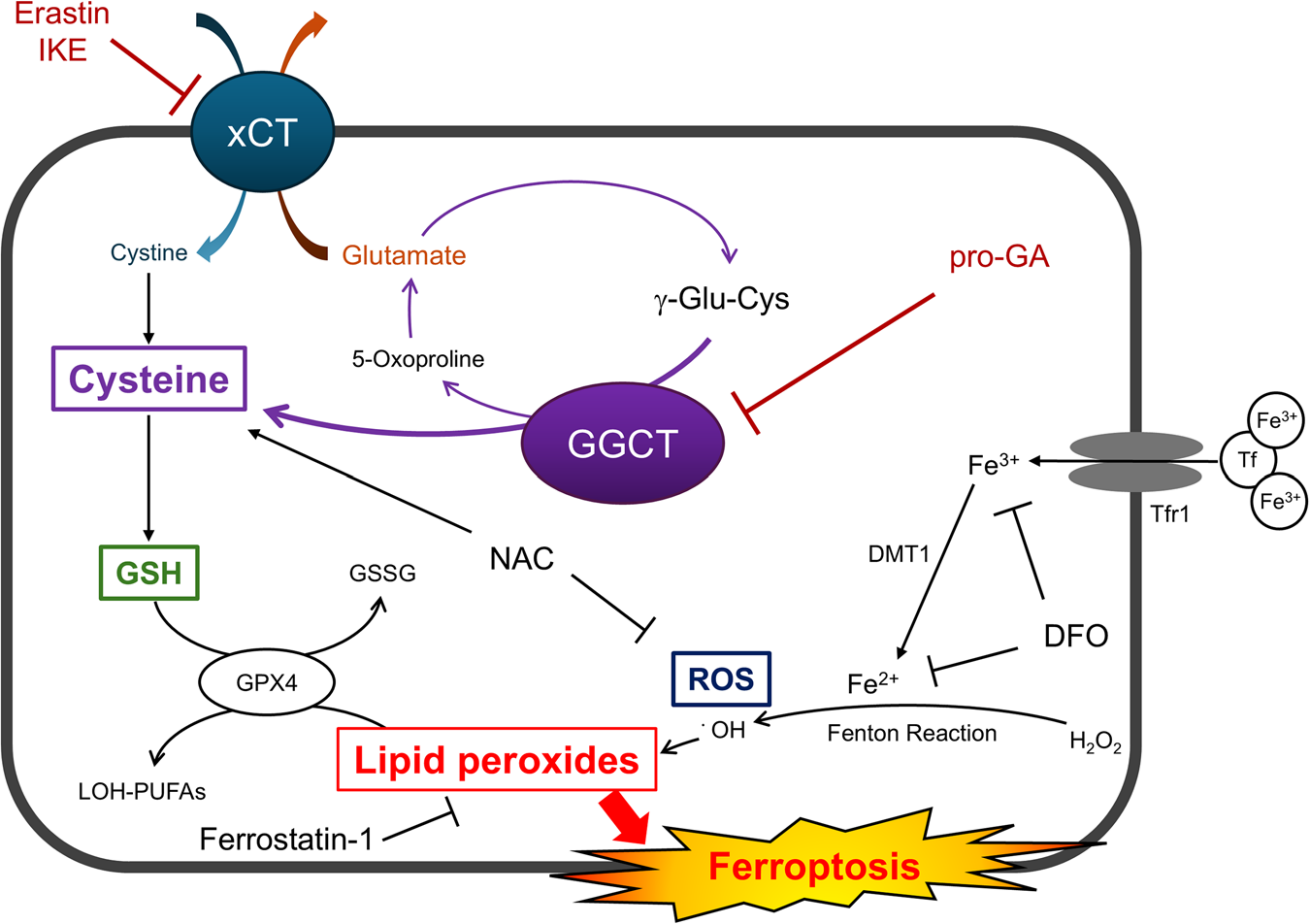
Schematic illustration of the mechanism by which combined inhibition of GGCT and xCT induces ferroptosis in glioblastoma cells. The xCT antiporter imports cystine in exchange for glutamate, supplying intracellular cysteine required for glutathione (GSH) synthesis. GGCT catalyzes the degradation of GSH, thereby recycling its constituent amino acids, including cysteine. Dual inhibition of xCT (by erastin or imidazole ketone erastin [IKE]) and GGCT (by pro-GA) depletes intracellular cysteine and GSH, leading to lipid peroxide accumulation and subsequent ferroptotic cell death.

Collectively, these results show that inhibiting both GGCT and xCT together effectively blocks the supply of intracellular cysteine, thereby inducing ferroptosis via GSH depletion and redox collapse, which in turn suppresses glioblastoma cell proliferation (Fig. 4).

### Dual inhibition of GGCT and xCT enhances antitumor efficacy in vivo without systemic toxicity

**Fig. 5:**
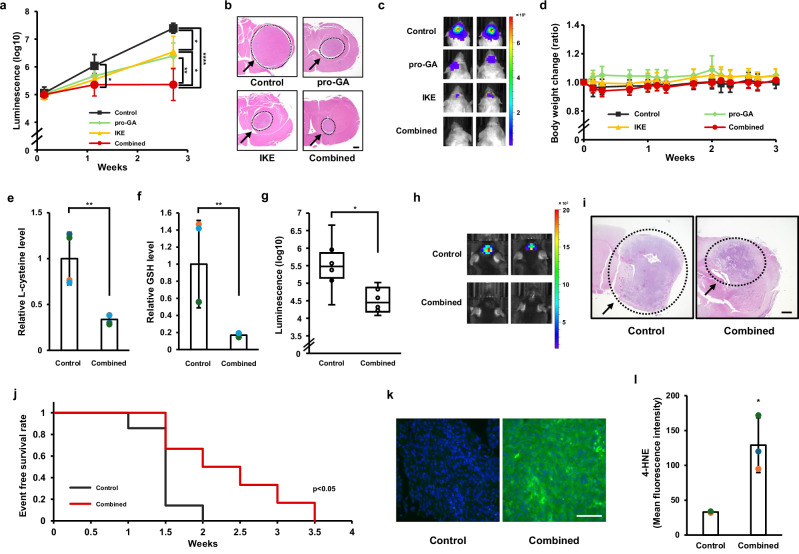
Dual inhibition of GGCT and xCT induces ferroptosis and exerts antitumor effects against glioblastoma in vivo. **a** Tumor growth in an orthotopic glioblastoma mouse model treated with pro-GA, imidazole ketone erastin (IKE), or a combination of the two. Bioluminescence imaging data were collected on Days 1, 8, and 19 after cell transplantation (Control, pro-GA: n = 5; IKE, combination: n = 6; ANOVA with Bonferroni’s multiple comparison test). **b** Representative images of hematoxylin and eosin (H&E)-stained brain sections at 3 weeks after transplantation. Scale bar: 500 μm. **c** Representative bioluminescence images of mice treated with control, pro-GA, IKE, or their combination on Day 19 after transplantation. **d** Changes in body weight were monitored throughout the experimental period (Control, pro-GA: n = 5; IKE, combination: n = 6; one-way ANOVA with Dunnett’s multiple comparison test vs. control). Intratumoral L-cysteine (**e**) and reduced GSH (**f**) levels in brain tumor tissues derived from U87MG orthotopic mouse models were measured by LC–MS/MS. Values were normalized to the control brain tissue group (control, n = 4; combined, n = 4). **g** Tumor growth in an orthotopic glioblastoma mouse model treated with pro-GA, IKE, or their combination. Bioluminescence imaging data were collected on Day 14 after cell transplantation (Control: n = 8; IKE: n = 7; pro-GA, combination: n = 6; Steel–Dwass test for multiple comparisons). **h** Representative bioluminescence images of mice treated with control or the combination of pro-GA and IKE on Day 14 after transplantation. **i** Representative images of H&E-stained brain sections on Day 14 after transplantation. Scale bar: 500 μm. **j** Event-free survival of mice orthotopically transplanted with GSC and treated intraperitoneally with the indicated compounds was analyzed using the Kaplan–Meier method. **k** Ferroptosis induction was assessed by immunofluorescence staining of 4-hydroxynonenal (4-HNE), a marker of lipid peroxidation. Representative images show increased green fluorescence signals in the combination therapy group. **l** Quantitative analysis of 4-HNE fluorescence intensity per cell shows significantly higher intensity in the combination group compared with the control (n = 3; ANOVA with Bonferroni’s multiple comparison test). Data are presented as mean ± SD. *p < 0.05, **p < 0.01, ****p < 0.0001.

To evaluate the therapeutic efficacy of dual GGCT and xCT inhibition in glioblastoma, we orthotopically implanted U87MG cells into the cerebral cortex of immunodeficient mice and treated them with pro-GA, the xCT inhibitor imidazole ketone erastin (IKE), or their combination. Bioluminescence imaging revealed that tumor growth in the combination group was significantly suppressed from Week 1 post-implantation and remained inhibited through Week 2.5, showing a marked reduction compared with the control and monotherapy groups (Fig. 5a). Hematoxylin and eosin (H&E) staining at the site of the largest tumor diameter demonstrated pronounced tumor regression in the combination therapy group, exceeding the effects observed with single-agent treatment (Fig. 5b). IVIS imaging further confirmed the reduced tumor burden in mice receiving the combination therapy (Fig. 5c).

Throughout the treatment period, no significant changes in body weight were observed among the groups (Fig. 5d). Moreover, H&E staining of liver and kidney tissues revealed no histopathological abnormalities (Fig. S3), indicating that the combination regimen caused no overt systemic toxicity.

To determine whether cysteine/GSH depletion also occurs in vivo, we performed LC–MS/MS analysis of tumor-bearing brain tissues obtained from U87MG xenograft mice. The combination treatment group exhibited significantly reduced levels of intratumoral cysteine (Fig. 5e) and GSH (Fig. 5f) compared with the control group. These findings indicate that the dual GGCT and xCT inhibition disrupts cysteine/GSH homeostasis within tumor tissues in vivo.

We next assessed therapeutic efficacy in an immunocompetent mouse model by orthotopic transplantation of GSC1 cells into the cerebral cortex of C57BL/6 mice. Quantitative bioluminescence analysis at Week 1 revealed a significant reduction in tumor burden in the combination group compared with the control (Fig. 5g), which was consistent with the IVIS imaging results (Fig. 5h). H&E staining again confirmed marked tumor regression in the combination group (Fig. 5i). Kaplan–Meier survival analysis showed that dual inhibition of GGCT and xCT significantly prolonged survival relative to that in the control group (Fig. 5j).

To determine whether ferroptosis contributed to these antitumor effects in vivo, we performed immunofluorescence staining for 4-hydroxynonenal (4-HNE), a marker of lipid peroxidation, in GSC1-derived tumor tissues. Fluorescence intensity was elevated in the treated groups, with the strongest signal observed in the combination therapy group (Fig. 5k). Quantitative analysis confirmed a significant increase in 4-HNE fluorescence in the combination group compared with the control (Fig. 5 l).

In addition, quantitative analysis confirmed detectable levels of GA (the active metabolite of pro-GA) and IKE in tumor-bearing brain tissues, with measurable tumor-to-serum and tumor-to-brain ratios (Fig. S4a, b).

Collectively, these findings demonstrate that dual inhibition of GGCT and xCT effectively suppresses glioblastoma growth in vivo, including that of therapy-resistant GSC, by inducing ferroptosis without causing apparent systemic toxicity.

## Discussion

Cancer cells are exposed constantly to high levels of oxidative stress, yet they survive by utilizing robust antioxidant defense mechanisms. GSH serves as a primary intracellular antioxidant, and cysteine availability plays a critical role in maintaining GSH levels, as supply of cysteine is the rate-limiting step in GSH synthesis [16, 26]. Previous studies show that cystine uptake from the extracellular environment via xCT is essential for maintenance of GSH homeostasis. In this study, we found that a supply of endogenous cysteine through GGCT may complement this GSH maintenance system. These findings may lead to development of novel strategies that target the antioxidant defense system of cancer cells.

The results of this study indicate that the salvage pathway for cysteine supply via GGCT helps to maintain GSH levels and serves as a resistance mechanism against therapeutic strategies that block cystine uptake via xCT inhibition. Indeed, previous reports show that some cancer cells can survive xCT inhibition alone [27], and our data support these findings. Therefore, we hypothesized that simultaneous inhibition of GGCT, which is highly expressed in various cancer cells, may overcome this resistance and increase the depletion of GSH, thereby providing a more cancer-specific therapeutic approach. Recent studies report direct transcriptional upregulation of GGCT by oncogenes such as c-Myc and c-Jun, which are considered undruggable; these findings suggest that inhibiting GGCT may be effective in cancers driven by these oncogenic factors [17, 28]. Additionally, previous studies report that GGCT inhibitor have minimal impact on normal cells [24], and that GGCT knockout mice grow almost normally and are able to reproduce [19, 28]. Notably, treatment with either pro-GA, erastin, or their combination did not affect the viability of hPBMCs, indicating that the dual inhibition selectively targets glioblastoma cells while sparing normal cells. This cancer cell–specific effect supports the therapeutic potential of this strategy with minimal systemic toxicity.

Treatment with the GGCT inhibitor pro-GA increased depletion of intracellular cysteine and GSH induced by the xCT inhibitor erastin, leading to ferroptosis accompanied by accumulation of lipid peroxides (Fig. 3). Previous reports show that depleting GSH promotes ferroptosis in cancer cells harboring Ras mutations [13], which is consistent with our findings. Ferroptosis induced by the combined inhibition of GGCT and xCT was suppressed by the ferroptosis inhibitor Fer-1. Moreover, NAC suppressed ferroptosis, indicating that the antitumor effect of dual GGCT and xCT inhibition is primarily driven by an increase in ROS caused by depletion of cysteine and GSH.　In addition to cysteine and GSH depletion, our data showed a marked increase in total and mitochondrial ROS following dual inhibition, which was effectively reversed by NAC treatment. These findings further confirm that redox collapse and oxidative stress play a central role in ferroptosis induced by the combined treatment.

Data from a glioblastoma mouse model showed that combined therapy with pro-GA and IKE led to a significant reduction in tumor volume without evident systemic toxicity. In our experiments, erastin did not show significant efficacy in vivo (data not shown). Other studies also report that, despite its strong ferroptosis-inducing effects in vitro, erastin exhibits limited in vivo efficacy due to its rapid metabolism and degradation. By contrast, IKE, which retains the properties of erastin but has better stability, demonstrates robust anti-numerable activity in vivo [29]. In vivo studies demonstrated tumor-specific effects, suggesting that dual inhibition of GGCT and xCT may be a promising new therapeutic strategy.

Although GA (the active form of pro-GA) and IKE were detectable in tumor-bearing brain tissues following systemic administration, optimization of drug delivery across the blood–brain barrier (BBB) remains an important consideration for further translational research. Recent advances in ferroptosis-amplifying nanotechnologies, including single-atom nanozyme systems, metal–polyphenol nanostructures, and glutathione-responsive designs, have demonstrated that modulation of redox homeostasis can enhance ferroptosis-based tumor therapy [30–33]. While our study employs a small-molecule-based strategy targeting cysteine metabolism via dual GGCT/xCT inhibition, the concept of GSH depletion-mediated ferroptosis is mechanistically aligned with these approaches. Integration with advanced delivery platforms and comprehensive pharmacokinetic evaluation will be important future directions.

Cancer stem cells (CSC) maintain low ROS status, and their enhanced antioxidant system contributes to therapy resistance [34]. Thus, CSCs evade conventional therapies by sustaining their antioxidant environment; therefore, disrupting this environment may improve therapeutic efficacy. Our data demonstrate that dual inhibition of GGCT and xCT disrupts the antioxidant control mechanisms that maintain the low ROS status of CSCs, which aligns with the above-mentioned concept and presents a promising strategy for overcoming therapy resistance. Conventional radiotherapy and chemotherapy have limited efficacy against CSCs [35]; however, our findings suggest that dual inhibition approaches may effectively counteract CSC resistance.

Future research should further explore the clinical applicability of dual inhibition of GGCT and xCT. Given the distinct mechanisms of this strategy, combining it with existing chemotherapy or immunotherapy is expected to enhance therapeutic efficacy further. Targeting the antioxidant stress response could help to overcome resistance to conventional therapies [36]; however, this approach remains insufficiently validated. Additionally, establishment of detailed optimal drug administration conditions for GGCT and xCT inhibitors will be an important step, although further research is required to advance this strategy.

Thus, it remains to be determined whether the therapeutic efficacy observed here can be generalized across diverse patient populations. Addressing these limitations will be crucial in translating this approach into a clinically viable therapy.

In summary, we demonstrate that the antioxidant defense system of glioblastoma cells relies not only on a supply of exogenous cysteine from cystine via xCT, but also on an endogenous supply of cysteine via GGCT. Blockade of cysteine availability through dual inhibition of GGCT and xCT effectively depletes GSH from glioblastoma cells. Addition of GGCT inhibition specifically targets the intrinsic mechanisms that enable glioblastoma cells to resist xCT inhibition, thereby providing a novel therapeutic strategy to efficiently induce ferroptosis in this highly aggressive and devastating malignancy.

## Materials and methods

### Reagents

The GGCT inhibitor pro-GA (FDV-0019, Funakoshi, Tokyo, Japan), erastin (S7242, Selleck Chemicals), imidazole ketone erastin (IKE; S8877, Selleck Chemicals, Houston, TX, USA), ferrostatin-1 (SML0583, Sigma-Aldrich, St. Louis, MO, USA), and deferoxamine mesylate (DFO; 252750, Sigma-Aldrich) were dissolved in dimethyl sulfoxide (DMSO; Nacalai Tesque, Kyoto, Japan). N-acetyl-L-cysteine (013-05133, FUJIFILM Wako, Osaka, Japan) was dissolved in phosphate-buffered saline (PBS).

### Cell culture and dye exclusion assay

Human glioblastoma cell lines U87MG, U251, A172, and T98 were obtained from the American Type Culture Collection (Manassas, VA, USA). Luciferase-expressing U87MG (Luc-U87MG) cells were established using pBLIV713PA-1 (System Biosciences, Palo Alto, CA, USA) followed by puromycin selection (125 ng/mL). Cells were cultured in DMEM (FUJIFILM Wako) supplemented with 10% fetal bovine serum (FBS) and 1% penicillin/streptomycin (FUJIFILM Wako). Cells were seeded in six-well plates, cultured overnight, and treated for 3 days with pro-GA and/or erastin at the indicated concentrations.

Primary murine glioblastoma cell lines (GSC1, GSC2, and GSC3) were independently established from separate mice that developed glioblastoma using the *Sleeping Beauty* transposon system, as previously described [37]. Sphere cultures derived from this primary cell line were maintained using the neurosphere method as described previously [32]. Glioblastoma stem cells (GSC) were treated with drugs immediately after seeding in six-well plates and cultured for 3 days.

Human peripheral blood mononuclear cells (hPBMCs) were isolated from healthy donor blood using density-gradient centrifugation with Lymphoprep™ (#07801, STEMCELL Technologies, Vancouver, Canada) according to the manufacturer’s instructions. Briefly, whole blood was diluted 1:1 with PBS containing 2% FBS, layered over Lymphoprep™, and centrifuged at 800 × *g* for 20 min at room temperature with the brake off. The mononuclear cell layer was collected and washed once with PBS + 2% FBS. Cells were resuspended in RPMI-1640 medium supplemented with 10% FBS and 1% penicillin/streptomycin, seeded into six-well plates, and treated with the indicated compounds for 3 days.

Cells were dissociated with trypsin-EDTA (FUJIFILM Wako) or Accutase (Innovative Cell Technologies, San Diego, CA, USA), stained with 0.4% Trypan Blue, and viable cells were counted using a Countess II automated cell counter (Thermo Fisher Scientific, Waltham, MA, USA).

### Detection of lipid peroxidation and reactive oxygen species by flow cytometry

Lipid peroxidation in cell membranes was assessed using BODIPY 581/591 C11 (Thermo Fisher Scientific), a fluorescent probe widely used to detect ferroptosis-associated lipid peroxidation. U87MG cells and GSCs were seeded in six-well plates and treated for 3 days with pro-GA, erastin, or ferrostatin-1 at the indicated concentrations. After treatment, cells were incubated with BODIPY 581/591 C11 (2 µM) for 30 min at 37 °C.

In parallel, total intracellular reactive oxygen species (ROS) levels were measured using the ROS Assay Kit (Highly Sensitive DCFH-DA; D6883, Dojindo, Kumamoto, Japan), and mitochondrial ROS levels were detected with MitoROS™ 580 (16052, AAT Bioquest, Sunnyvale, CA, USA). Cells were incubated with each probe under the manufacturer’s recommended conditions following 3-day drug treatment. After staining, cells were dissociated, washed with PBS, and resuspended in 500 µL of PBS. Flow cytometric analysis was performed using a BD LSRFortessa™ X-20 (BD Biosciences, Franklin Lakes, NJ, USA). A minimum of 10,000 events per sample was analyzed.

### Western blotting

Cells were lysed with 1% sodium dodecyl sulfate (SDS) buffer containing 50 mM Tris-HCl (pH 7.5), protease inhibitor cocktail (Nacalai Tesque), and PhosSTOP EASYpack (Roche Diagnostics, Mannheim, Germany). Protein concentration was measured using a BCA Protein Assay Kit (Thermo Fisher Scientific). Equal amounts of protein (20 µg) were separated by SDS-PAGE on 12% polyacrylamide gels and transferred onto polyvinylidene difluoride membranes (Merck Millipore, Darmstadt, Germany).

Membranes were blocked with 3% BSA in TBS-T for detection of GGCT and β-actin, or with 3% skim milk in TBS-T for xCT detection. For ACSL4, LPCAT3, and GPX4 detection, membranes were blocked with 5% skim milk in TBS-T. The membranes were incubated with the following primary antibodies: GGCT (ab198503, 1:500, Abcam, Cambridge, UK), xCT (ab238078, 1:500, Abcam), β-actin (M177-3, 1:2,000, MBL, Tokyo, Japan), ACSL4 (F6T3Z, #38493, 1:1000, Cell Signaling Technology, Danvers, MA, USA), LPCAT3 (EPR28798-172, 1:1000, Abcam), and GPX4 (ab125066, 1:1000, Abcam), followed by secondary antibodies. Chemiluminescent signals were detected using Clarity Western ECL Substrate (Bio-Rad Laboratories, Hercules, CA, USA) and imaged with a ChemiDoc XRS Plus system (Bio-Rad). Secondary antibodies used were horse anti-mouse IgG-HRP (PI-2000, 1:5000, Vector Laboratories, Burlingame, CA, USA) and HRP-linked goat anti-rabbit IgG (#7074, 1:2000, Cell Signaling Technology, Danvers, MA, USA).

### Glutathione peroxidase activity assay

Total GPx activity was measured using a colorimetric Glutathione Peroxidase Assay Kit (ab102530; Abcam, Cambridge, UK) according to the manufacturer’s instructions. Briefly, 2 × 10^6^ cells were harvested and homogenized in the assay buffer provided in the kit. After centrifugation at 10,000 × *g* for 15 min at 4 °C, the supernatant was collected and used for analysis. GPx activity was determined by monitoring the decrease in NADPH absorbance at 340 nm for 5 min at 25 °C using a microplate reader. All samples were prepared using an equal number of cells. Enzyme activity was calculated based on the change in absorbance (ΔA340 nm) and expressed relative to the control group, which was set to 1.

### LC-MS/MS analysis

The levels of cysteine and GSH in vitro and in vivo, and GA and IKE in vivo, were measured on a LC-MS/MS system as described previously, with minor modifications [29, 38–40]. Briefly, a Shimadzu LCMS-8040 (Triple Quadrupole Mass Spectrometer, Shimadzu) with an electrospray ionization (ESI) source was used for analysis. Test samples were separated on a YMC-Triart C18 column (50 × 2.0 mm i.d.; S-3 µm; YMC) at a flow rate of 0.2 mL/min at 40 °C. The mobile phases were water/0.2% formic acid (A) and acetonitrile (B). Gradient elution was performed using the following program: 0–5 min 5% B, 5–10 min 5%–95% B, 10–12 min 95%–5% B. The injected volume of test samples was 10 µL. For in vitro experiments, samples were processed by suspending cells at a concentration of 5 × 10⁶/mL in 50% methanol. The suspension was subjected to repeated freeze–thaw cycles using liquid nitrogen, followed by centrifugation. The resulting supernatant was passed through a 0.22 µm filter before analysis. For in vivo experiments, U87MG tumor-bearing mice were intraperitoneally administered pro-GA (25 mg/kg) or IKE (25 mg/kg) three times per week for three weeks for tissue distribution analysis. Vehicle-treated mice were processed in parallel. In both experiments, tissues were collected 1 h after the final administration. Brain tumors, normal brain tissue, and serum were harvested, homogenized in 50% methanol, and processed as described above. Protein concentrations of tissue samples were determined using a BCA protein assay, and measured analyte levels were normalized to total protein content. Calibration standards were prepared by diluting L-cysteine reduced GSH in 50% methanol. The conditions for interface were as follows: DL temperature, 250 °C; nebulizing gas flow, 3 L/min; heat block temperature, 400 °C; drying gas flow, 15 L/min; and Interface Voltage, 4.5 kV. The following MRM pairs were monitored in positive mode: *m/z* 122.10 → 59.05 (CE 24 eV) for L-cysteine, and 308.10 → 179.05 (CE 14 eV) for reduced GSH; and m/z 204.20 → 90.10 for GA. IKE was monitored in positive ion mode at m/z 655.40. The dwell time was 100 ms.

### Antitumor effects in a glioblastoma mouse model

All experiments were conducted in accordance with guidelines approved by the Animal Experimentation Ethics Committee of Kyoto Pharmaceutical University (Approval No.: A21-011-02). For the U87MG model, male CB-17 severe combined immunodeficient (SCID) mice (8 weeks old, n = 5 or 6 per group) were purchased from Japan SLC (Shizuoka, Japan). A cell suspension containing 5 × 10^4^ Luc-U87MG cells in 2 μL of PBS (FUJIFILM Wako) was prepared an injected into the right cerebrum (under anesthesia) using an infusion system (Legato 130) and a flow rate of 1 μL/min; the injection site was located 2 mm lateral and 3 mm ventral from the bregma. Starting 2 days post-transplantation, mice were injected intraperitoneally, three times per week, with DMSO, imidazole ketone erastin (IKE; 25 mg/kg), pro-GA (25 mg/kg), or a combination of IKE plus pro-GA. IKE or pro-GA were dissolved at 100 mg/mL or 200 mg/mL in DMSO with Kolliphor EL (Sigma-Aldrich; final concentration, 10%) and then diluted in saline (total 100 μL/20 g body weight) to the required concentration. DMSO was used as a control. An IVIS Lumina XR imaging system was used to measure bioluminescence intensity 10 min after injection of D-luciferin (Wako Pure Chemical Industries, Osaka, Japan). Additionally, the body weight of each mouse was measured four times per week to monitor potential treatment-related toxicity. For the GSC1 model, wild-type C57BL/6 mice (8 weeks old, n = 6, 7 or 8 per group) were obtained from Oriental BioService (Kyoto, Japan). A cell suspension containing 1000 Luc-GSC1 cells in 2 μL of PBS was injected orthotopically into the right cerebrum under the same conditions used for the U87MG model (i.e., an infusion rate of 1 μL/min, and an injection site 2 mm lateral and 3 mm ventral from the bregma). Intraperitoneal administration of DMSO, IKE (25 mg/kg), pro-GA (25 mg/kg), or a combination of the two, was started 1 week after transplantation, and conducted three times per week using the same formulation and dosing method described above. Bioluminescence imaging was performed as described for the U87MG model. Animals were allocated to treatment groups after tumor implantation without formal randomization. Sample sizes were not determined by a formal power calculation; instead, the minimum number of animals required to obtain reliable results was used in accordance with the 3R principles (Replacement, Reduction, and Refinement).

### Immunohistochemical and hematoxylin and eosin (H&E) staining

Tissues were fixed with phosphate buffered 10% formalin and then embedded in paraffin. Paraffin-embedded sections (4 μm) were deparaffinized in xylene and rehydrated through descending concentrations of ethanol. Antigen retrieval was performed using TE buffer (50 mM Tris, 0.2 mM EDTA, pH 9.0) and heating for 40 min in a steam cooker. For blocking, sections were incubated with 3% BSA at room temperature for 1 h and then overnight at 4 °C with an anti-4-hydroxynonenal (4-HNE) antibody (MAB3249, R&D Systems, Minneapolis, MN, USA; 0.1 µg/mL) diluted with antibody diluent containing background reducing components (S3022; Dako Agilent pathology Solutions, Santa Clara, CA, USA). Sections were washed three times with TBS and incubated at room temperature for 1 h with Polyclonal Rabbit Anti-Mouse Immunoglobulins/FITC (F0261; Dako Agilent Pathology Solutions). After washing three times with TBS, the sections were stained for 5 min at room temperature with DAPI Nucleic Acid Stain (PA-3013, Lonza, Basel, Switzerland) and washed with TBS. Finally, the sections were mounted using VectaMount AQ Aqueous Mounting Medium (Vector Laboratories, Newark, CA, USA). Fluorescent signals were detected under an All-in-one fluorescence microscope (BZ-X800, Keyence, Osaka, Japan), and the mean fluorescence intensity of 4-HNE per cell was measured using the BZ-X800 Wide Image Viewer analysis software. For H&E staining, nuclei were stained for 4 min with Mayer’s hematoxylin solution (131-09665, FUJIFILM Wako) and then rinsed under running water for 5 min to develop the color. The cytoplasm was then stained for 1 min with 1% Eosin Y Solution (051-06515, FUJIFILM Wako). After staining, the sections were dehydrated with xylene, and mounted using Mount-Quick (Daido Sangyo, Saitama, Japan).

### Statistical analysis

Data were collected from a minimum of three independent experiments, and are presented as the mean ± SD. Statistical comparisons were conducted using ANOVA with Bonferroni’s multiple comparison test, Dunnett’s multiple comparison test or Steel–Dwass test. All analyses were performed using Bell Curve for Excel (Social Survey Research Information Co., Ltd., Tokyo, Japan). The synergistic effects of pro-GA and erastin on cell growth inhibition were assessed using the isobologram and combination index (CI; CalcuSyn 2.11 software; Biosoft, Cambridge, UK). A CI value of <0.9, from 0.9–1.1, or > 1.1 was considered as synergy, additivity, or antagonism, respectively. For the in vivo survival analysis, Kaplan–Meier survival curves were generated, and statistical significance was assessed using the log-rank test. For event-free survival analysis, an event was defined as either tumor signal exceeding 1.0 × 10^6^ photons/s as measured by IVIS, or body weight loss greater than 20% from the initial body weight. An event was considered to have occurred if either criterion was met, and Kaplan–Meier analysis was performed accordingly. Notably, no mice met the event criteria due to body weight loss. Investigators were not blinded to treatment allocation during experiments or outcome assessment. A *p*-value < 0.05 was considered statistically significant (*p < 0.05; **p < 0.01; ***p < 0.001; and ****p < 0.0001).

## Supplementary information

Supplementary Legends

Supplementary_Figure_1

Supplementary_Figure_2

Supplementary_Figure_3

Supplementary_Figure_4

Supplementary_Original_WB

## Data Availability

The data generated in this study are available from the corresponding author upon reasonable request.

## References

[1] Louis DN, Perry A, Reifenberger G, von Deimling A, Figarella-Branger D, Cavenee WK, et al. The 2016 World Health Organization classification of tumors of the central nervous system: a summary. Acta Neuropathol. 2016;131:803–20. 27157931 10.1007/s00401-016-1545-1

[2] Stupp R, Mason WP, van den Bent MJ, Weller M, Fisher B, Taphoorn MJ, et al. Radiotherapy plus concomitant and adjuvant temozolomide for glioblastoma. N Engl J Med. 2005;352:987–96. 15758009 10.1056/NEJMoa043330

[3] Tan AC, Ashley DM, López GY, Malinzak M, Friedman HS, Khasraw M. Management of glioblastoma: state of the art and future directions. CA Cancer J Clin. 2020;70:299–312. 32478924 10.3322/caac.21613

[4] Johnson DR, Leeper HE, Uhm JH. Glioblastoma survival in the United States improved after Food and Drug Administration approval of bevacizumab. Cancer. 2013;119:3489–95. 23868553 10.1002/cncr.28259

[5] Lathia JD, Heddleston JM, Venere M, Rich JN. Deadly teamwork: neural cancer stem cells and the tumor microenvironment. Cell Stem Cell. 2011;8:482–5. 21549324 10.1016/j.stem.2011.04.013 PMC3494093

[6] Singh SK, Hawkins C, Clarke ID, Squire JA, Bayani J, Hide T, et al. Identification of human brain tumour initiating cells. Nature. 2004;432:396–401. 15549107 10.1038/nature03128

[7] DeBerardinis RJ, Chandel NS. Fundamentals of cancer metabolism. Sci Adv. 2016;2:e1600200. 27386546 10.1126/sciadv.1600200 PMC4928883

[8] Lee J, Roh JL. SLC7A11 as a gateway of metabolic perturbation and ferroptosis vulnerability in cancer. Antioxidants. 2022;11:2444. 36552652 10.3390/antiox11122444 PMC9774303

[9] Jiang L, Kon N, Li T, Wang SJ, Su T, Hibshoosh H, et al. Ferroptosis as a p53-mediated activity during tumour suppression. Nature. 2015;520:57–62. 25799988 10.1038/nature14344 PMC4455927

[10] Stockwell BR, Friedmann Angeli JP, Bayir H, Bush AI, Conrad M, Dixon SJ, et al. Ferroptosis: a regulated cell death nexus linking metabolism, redox biology, and disease. Cell. 2017;171:273–85. 28985560 10.1016/j.cell.2017.09.021 PMC5685180

[11] Nagano O, Okazaki S, Saya H. Redox regulation in stem-like cancer cells by CD44 variant isoforms. Oncogene. 2013;32:5191–8. 23334333 10.1038/onc.2012.638

[12] Choi ES, Kim H, Kim HP, Choi Y, Goh SH. CD44v8-10 as a potential theranostic biomarker for targeting disseminated cancer cells in advanced gastric cancer. Sci Rep. 2017;7:4930. 28694503 10.1038/s41598-017-05247-7 PMC5503939

[13] Dixon SJ, Lemberg KM, Lamprecht MR, Skouta R, Zaitsev EM, Gleason CE, et al. Ferroptosis: an iron-dependent form of nonapoptotic cell death. Cell. 2012;149:1060–72. 22632970 10.1016/j.cell.2012.03.042 PMC3367386

[14] Yang WS, SriRamaratnam R, Welsch ME, Shimada K, Skouta R, Viswanathan VS, et al. Regulation of ferroptotic cancer cell death by GPX4. Cell. 2014;156:317–31. 24439385 10.1016/j.cell.2013.12.010 PMC4076414

[15] Ishimoto T, Nagano O, Yae T, Tamada M, Motohara T, Oshima H, et al. CD44 variant regulates redox status in cancer cells by stabilizing the xCT subunit of system xc- and thereby promotes tumor growth. Cancer Cell. 2011;19:387–400. 21397861 10.1016/j.ccr.2011.01.038

[16] Yang WS, Stockwell BR. Ferroptosis: death by lipid peroxidation. Trends Cell Biol. 2016;26:165–76. 26653790 10.1016/j.tcb.2015.10.014 PMC4764384

[17] Nose K, Taniguchi K, Fujita M, Moyama C, Mori M, Ishita M, et al. γ-Glutamylcyclotransferase is transcriptionally regulated by c-Jun and controls proliferation of glioblastoma stem cells through Notch1 levels. Cancer Gene Ther. 2024;31:57–66. 10.1038/s41417-024-00835-y 39394529

[18] Kageyama S, Ii H, Taniguchi K, Kubota S, Yoshida T, Isono T, et al. Mechanisms of tumor growth inhibition by depletion of γ-glutamylcyclotransferase (GGCT): a novel molecular target for anticancer therapy. Int J Mol Sci. 2018;19:2054. 30011933 10.3390/ijms19072054 PMC6073726

[19] He Z, Wang S, Shao Y, Zhang J, Wu X, Chen Y, et al. Ras downstream effector GGCT alleviates oncogenic stress. iScience. 2019;19:256–66. 31400748 10.1016/j.isci.2019.07.036 PMC6700472

[20] Oakley AJ, Yamada T, Liu D, Coggan M, Clark AG, Board PG. The identification and structural characterization of C7orf24 as γ-glutamylcyclotransferase, an essential enzyme in the γ-glutamyl cycle. J Biol Chem. 2008;283:22031–42. 18515354 10.1074/jbc.M803623200

[21] Matsumura K, Nakata S, Taniguchi K, Ii H, Ashihara E, Kageyama S, et al. Depletion of γ-glutamylcyclotransferase inhibits breast cancer cell growth via cellular senescence induction mediated by CDK inhibitor upregulation. BMC Cancer. 2016;16:748. 27658708 10.1186/s12885-016-2779-y PMC5034417

[22] Taniguchi K, Ii H, Kageyama S, Takagi H, Chano T, Kawauchi A, et al. Depletion of γ-glutamylcyclotransferase inhibits cancer cell growth by activating the AMPK–FOXO3a–p21 axis. Biochem Biophys Res Commun. 2019;517:238–43. 31345573 10.1016/j.bbrc.2019.07.049

[23] Ii H, Taniguchi K, Yoshiya T, Nohara Y, Kageyama S, Kawauchi A, et al. The γ-glutamylcyclotransferase inhibitor pro-GA induces an antiproliferative effect through the generation of mitochondrial reactive oxygen species. Anticancer Res. 2022;42:4311–7. 36039439 10.21873/anticanres.15931

[24] Ii H, Yoshiya T, Nakata S, Taniguchi K, Hidaka K, Tsuda S, et al. A novel prodrug of a γ-glutamylcyclotransferase inhibitor suppresses cancer cell proliferation in vitro and inhibits tumor growth in a xenograft mouse model of prostate cancer. ChemMedChem. 2018;13:155–63. 29316360 10.1002/cmdc.201700660

[25] Aoyama K, Watabe M, Nakaki T. Regulation of neuronal glutathione synthesis. J Pharmacol Sci. 2008;108:227–38. 19008644 10.1254/jphs.08r01cr

[26] Yoshikawa M, Tsuchihashi K, Ishimoto T, Yae T, Motohara T, Sugihara E, et al. xCT inhibition depletes CD44v-expressing tumor cells that are resistant to EGFR-targeted therapy in head and neck squamous cell carcinoma. Cancer Res. 2012;73:1855–66. 10.1158/0008-5472.CAN-12-3609-T 23319806

[27] Jyotsana N, Ta KT, DelGiorno KE. The role of cystine/glutamate antiporter SLC7A11/xCT in cancer cell survival and drug resistance. Front Oncol. 2022;12:858462. 35280777 10.3389/fonc.2022.858462 PMC8904967

[28] Ueno T, Otani S, Date Y, Katsuma Y, Nagayoshi Y, Ito T, et al. Myc upregulates Ggct, γ-glutamylcyclotransferase to promote development of p53-deficient osteosarcoma. Cancer Sci. 2024;115:2961–71. 38924236 10.1111/cas.16255 PMC11462974

[29] Zhang Y, Tan H, Daniels JD, Zandkarimi F, Liu H, Brown LM, et al. Imidazole ketone erastin induces ferroptosis and slows tumor growth in a mouse lymphoma model. Cell Chem Biol. 2019;26:623–33.e9. 30799221 10.1016/j.chembiol.2019.01.008 PMC6525071

[30] Zhu Y, Wang D, Du C, Wu T, Wei P, Zheng H, et al. Ruthenium single-atom nanozyme driven sonosensitizer with oxygen vacancies enhances electron-hole separation efficacy and remodels tumor microenvironment for sonodynamic-amplified ferroptosis. Adv Sci. 2025;12:e2416997. 10.1002/advs.202416997 PMC12165091 40279631

[31] Wu T, Wei P, Zhao P, Niu X, Ding C, Zhu Y. Engineering local coordination environment of manganese single-atom enzyme for amplified infected wound therapy. Chem Eng J. 2025;514:163247.

[32] Wu T, Niu X, Ding C, Fang W, Li T, Yan L, et al. Metal-polyphenol self-assembled nanodots for NIR-II fluorescence imaging-guided chemodynamic/photodynamic therapy-amplified ferroptosis. Acta Biomater. 2024;361–70. 10.1016/j.actbio.2024.07.017 39025392

[33] Wei P, Niu X, Wang D, Du C, Zhu M, Zheng H, et al. A glutathione-responsive ferroptotic inducer with elevated labile iron pool and self-supplied peroxide for chemodynamic therapy. Mater Today Biol. 2025;32:101913. 10.1016/j.mtbio.2025.101913 PMC12167041 40520549

[34] Diehn M, Cho RW, Lobo NA, Kalisky T, Dorie MJ, Kulp AN, et al. Association of reactive oxygen species levels and radioresistance in cancer stem cells. Nature. 2009;458:780–3. 19194462 10.1038/nature07733 PMC2778612

[35] Vlashi E, Pajonk F. Cancer stem cells, cancer cell plasticity and radiation therapy. Semin Cancer Biol. 2015;31:28–35. 25025713 10.1016/j.semcancer.2014.07.001 PMC4291301

[36] DeNicola GM, Karreth FA, Humpton TJ, Gopinathan A, Wei C, Frese K, et al. Oncogene-induced Nrf2 transcription promotes ROS detoxification and tumorigenesis. Nature. 2011;475:106–9. 21734707 10.1038/nature10189 PMC3404470

[37] Mori M, Ii H, Fujita M, Nose K, Shimada A, Shiraki R, et al. Desert Hedgehog down-regulation mediates inhibition of proliferation by γ-glutamylcyclotransferase knockdown in murine glioblastoma stem cells. Cancer Genom Proteom. 2024;21:474–84. 10.21873/cgp.20465 PMC11363923 39191500

[38] Guiraud SP, Montoliu I, Da Silva L, Dayon L, Núñez Galindo A, Corthésy J, et al. High-throughput and simultaneous quantitative analysis of homocysteine–methionine cycle metabolites and co-factors in blood plasma and cerebrospinal fluid by isotope dilution LC–MS/MS. Anal Bioanal Chem. 2017;409:295–305. 27757515 10.1007/s00216-016-0003-1 PMC5203846

[39] Dienes-Nagy Á, Vuichard F, Belcher S, Blackford M, Rösti J, Lorenzini F. Simultaneous quantification of glutathione, glutathione disulfide and glutathione-S-sulfonate in grape and wine using LC-MS/MS. Food Chem. 2022;386:132756. 35509159 10.1016/j.foodchem.2022.132756

[40] Ii H, Yoshiki T, Hoshiya N, Uenishi J. Synthesis and GGCT inhibitory activity of N-glutaryl-L-alanine analogues. Chem Pharm Bull. 2016;64:785–92. 10.1248/cpb.c16-00167 27373633

