# Supplementary material for: Dual inhibition of xCT and GGCT induces ferroptosis in glioblastoma cells by depleting cysteine and disrupting redox homeostasis
Source: Cell Death Discov. 2026 Apr 15;12:249. doi: 10.1038/s41420-026-03108-9 (PMC13201756; doi:10.1038/s41420-026-03108-9)
Supplement: Supplementary file 1 — Supplementary Legends [file 41420_2026_3108_MOESM1_ESM.docx]

**Supplementary Figure 1: Phase-contrast images of U87MG and GSC at the time of cell counting.**

Representative phase-contrast images of U87MG and GSC at the time of cell counting (related to Fig. 2a). U87MG cells were treated with control, pro-GA (60 µM), erastin (0.6 µM), or a combination of the two, with or without Ferrostatin-1 (Fer-1, 0 or 1 µM). GSC were treated with control, pro-GA (100 µM), erastin (0.1 µM), or a combination of the two, with or without Fer-1 (0 or 2 µM). Scale bar: 100 µm.

**Supplementary Figure 2: Evaluation of ferroptosis terminal execution mechanisms in U87MG cells and GSCs following dual inhibition.**

a: Western blot analysis of ACSL4, LPCAT3, and GPX4 protein levels after treatment with pro-GA, erastin, or their combination. b: Total GPX enzymatic activity under conditions supplemented with exogenous GSH.

**Supplementary Figure 3: Representative H&E-stained liver and kidney tissues from glioblastoma-bearing SCID mice**

Representative hematoxylin and eosin (H&E)-stained images of liver and kidney tissues (related to Fig. 4). H&E staining was performed on liver and kidney tissues collected from glioblastoma-bearing SCID mice treated with control, pro-GA (25 mg/kg), IKE (25 mg/kg), or a combination of the two. No histological abnormalities were observed in any of the groups. Scale bar: 100 µm.

**Supplementary Figure 4: Blood–brain barrier permeability of pro-GA and IKE in U87MG orthotopic mouse models.**

(a, b) Representative LC–MS/MS chromatograms of GA (a) and IKE (b) in brain tissues one hour after intraperitoneal administration. Clear peaks corresponding to each compound are indicated by arrows. Control samples show no detectable peaks. (c) Quantification of GA and IKE levels in tumor tissue, normal brain tissue, and serum (mean ± SD, n = 3). Ratios of tumor/serum, brain/serum, and tumor/brain are also shown, demonstrating measurable concentrations of both agents in the brain and tumor tissues, indicating their ability to penetrate the blood–brain barrier.
