# Supplementary figures and images for: Dual inhibition of xCT and GGCT induces ferroptosis in glioblastoma cells by depleting cysteine and disrupting redox homeostasis
Source: Cell Death Discov. 2026 Apr 15;12:249. doi: 10.1038/s41420-026-03108-9 (PMC13201756; doi:10.1038/s41420-026-03108-9)

Supplemental Figure 1

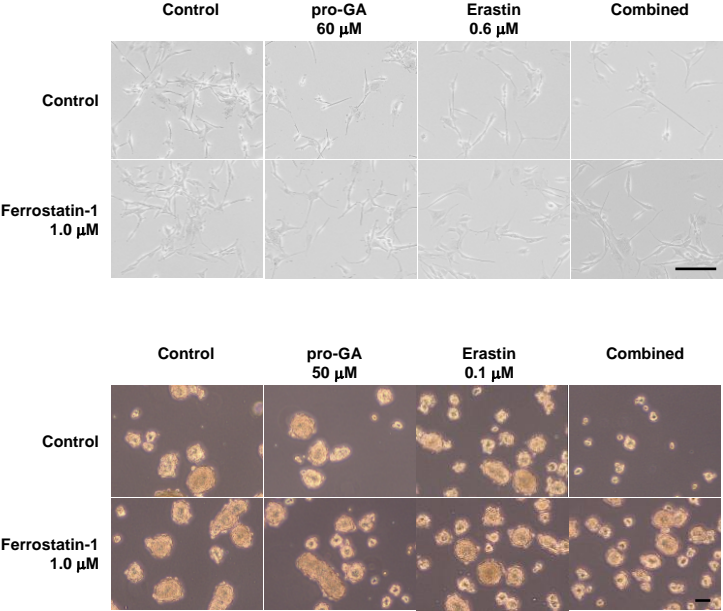

Supplement: Supplementary file 2 — Supplementary_Figure_1 [file 41420_2026_3108_MOESM2_ESM.pdf]

Supplemental Figure 2

a

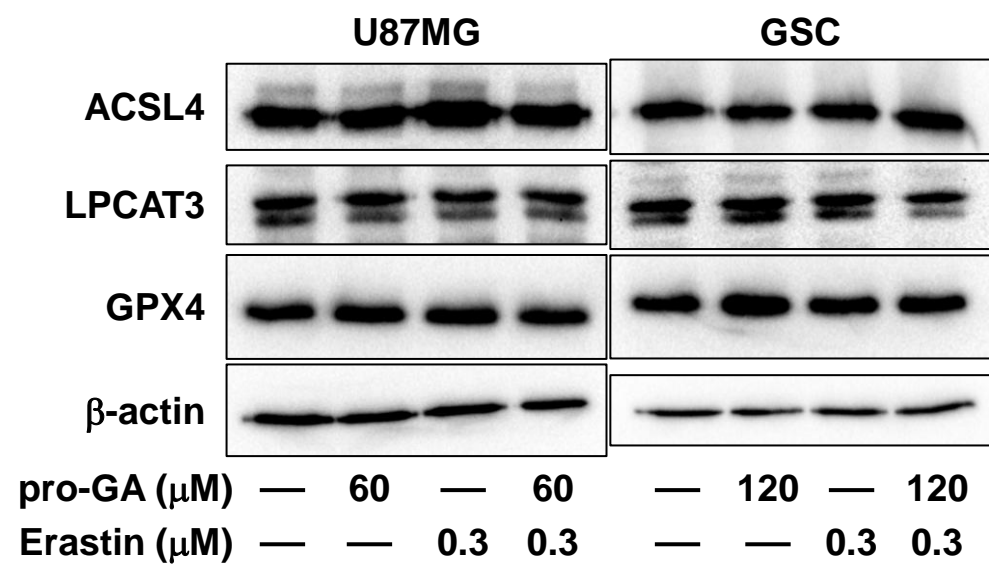

b

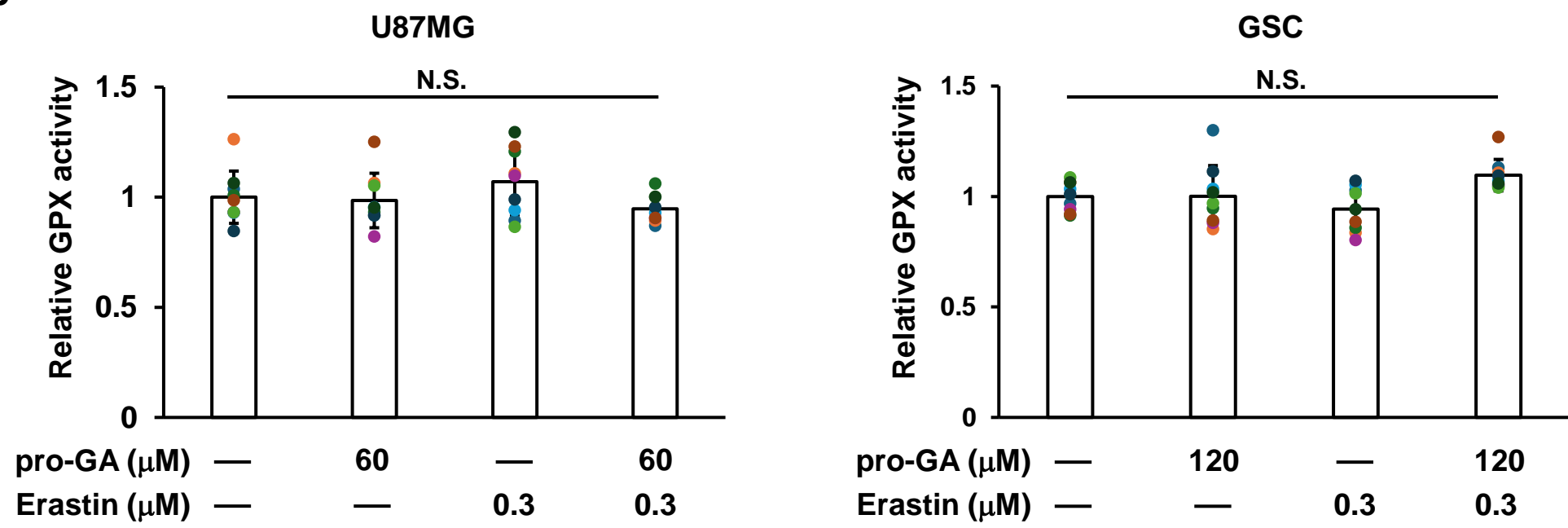

Supplement: Supplementary file 3 — Supplementary_Figure_2 [file 41420_2026_3108_MOESM3_ESM.pdf]

Supplemental Figure 3

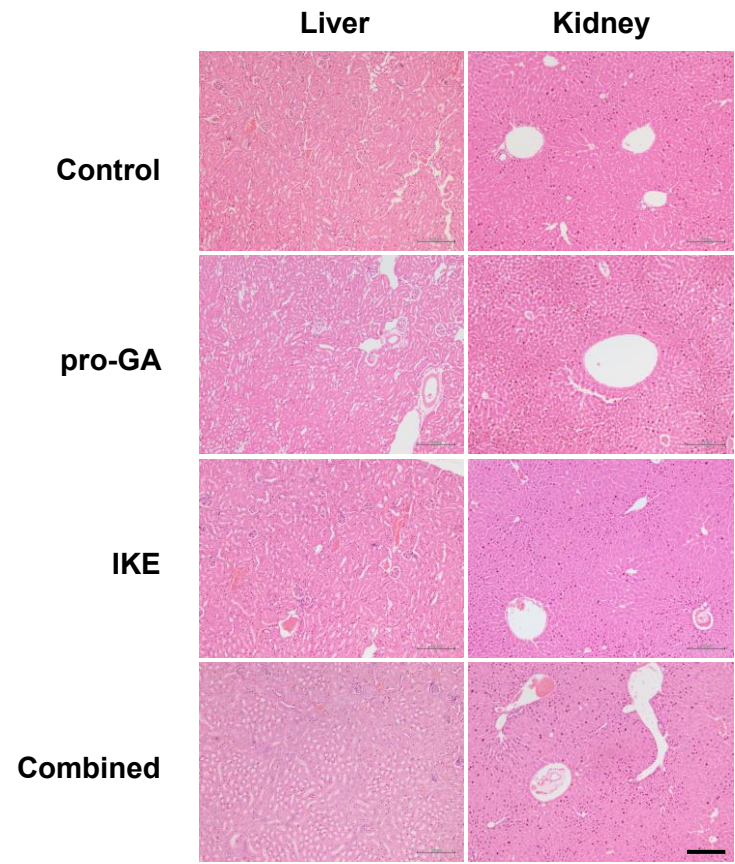

Supplement: Supplementary file 4 — Supplementary_Figure_3 [file 41420_2026_3108_MOESM4_ESM.pdf]
