# Supplementary material for: Dual inhibition of xCT and GGCT induces ferroptosis in glioblastoma cells by depleting cysteine and disrupting redox homeostasis
Source: Cell Death Discov. 2026 Apr 15;12:249. doi: 10.1038/s41420-026-03108-9 (PMC13201756; doi:10.1038/s41420-026-03108-9)
Supplement: Supplementary file 5 — Supplementary_Figure_4 [file 41420_2026_3108_MOESM5_ESM.pdf]

Supplemental Figure 4

a

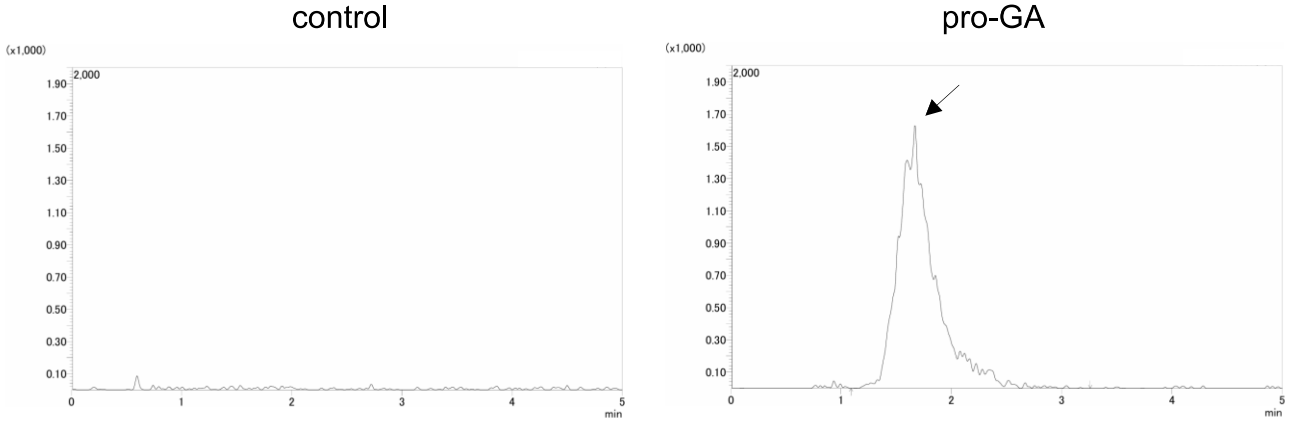

b

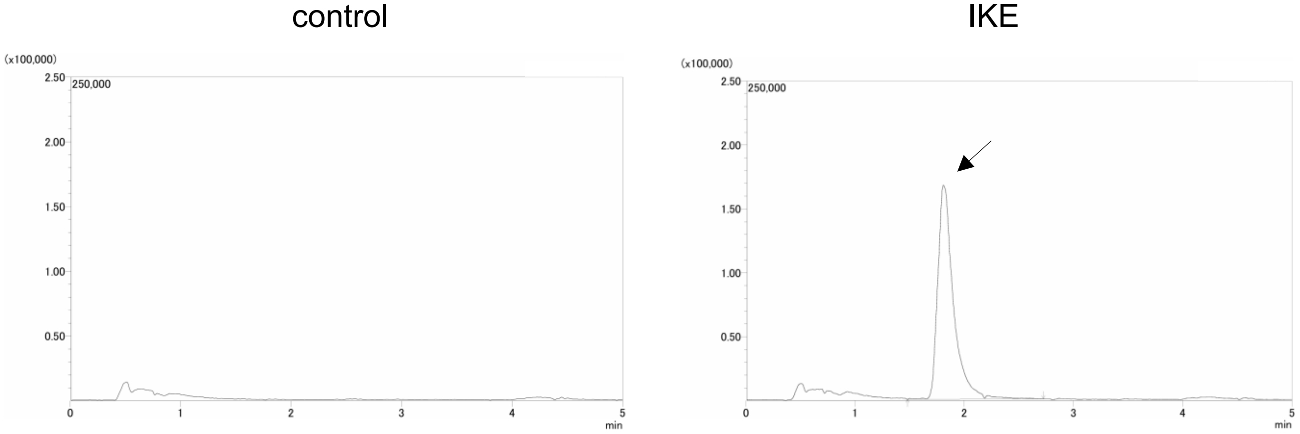

c

	GA (ng / mg protein)	IKE (ng / mg protein)
Tumor	71.79 ± 4.65	487.99 ± 244.48
Brain	55.76 ± 2.82	84.90 ± 14.09
Serum	463.95 ± 209.83	1614.19 ± 163.31

	GA	IKE
Tumor / Serum	0.16 ± 0.010	0.30 ± 0.15
Brain / Serum	0.12 ± 0.0061	0.053 ± 0.0087
Tumor / Brain	1.29 ± 0.083	5.75 ± 2.88
