# Supplementary material for: Dual inhibition of xCT and GGCT induces ferroptosis in glioblastoma cells by depleting cysteine and disrupting redox homeostasis
Source: Cell Death Discov. 2026 Apr 15;12:249. doi: 10.1038/s41420-026-03108-9 (PMC13201756; doi:10.1038/s41420-026-03108-9)
Supplement: Supplementary file 6 — Supplementary_Original_WB [file 41420_2026_3108_MOESM6_ESM.pdf]

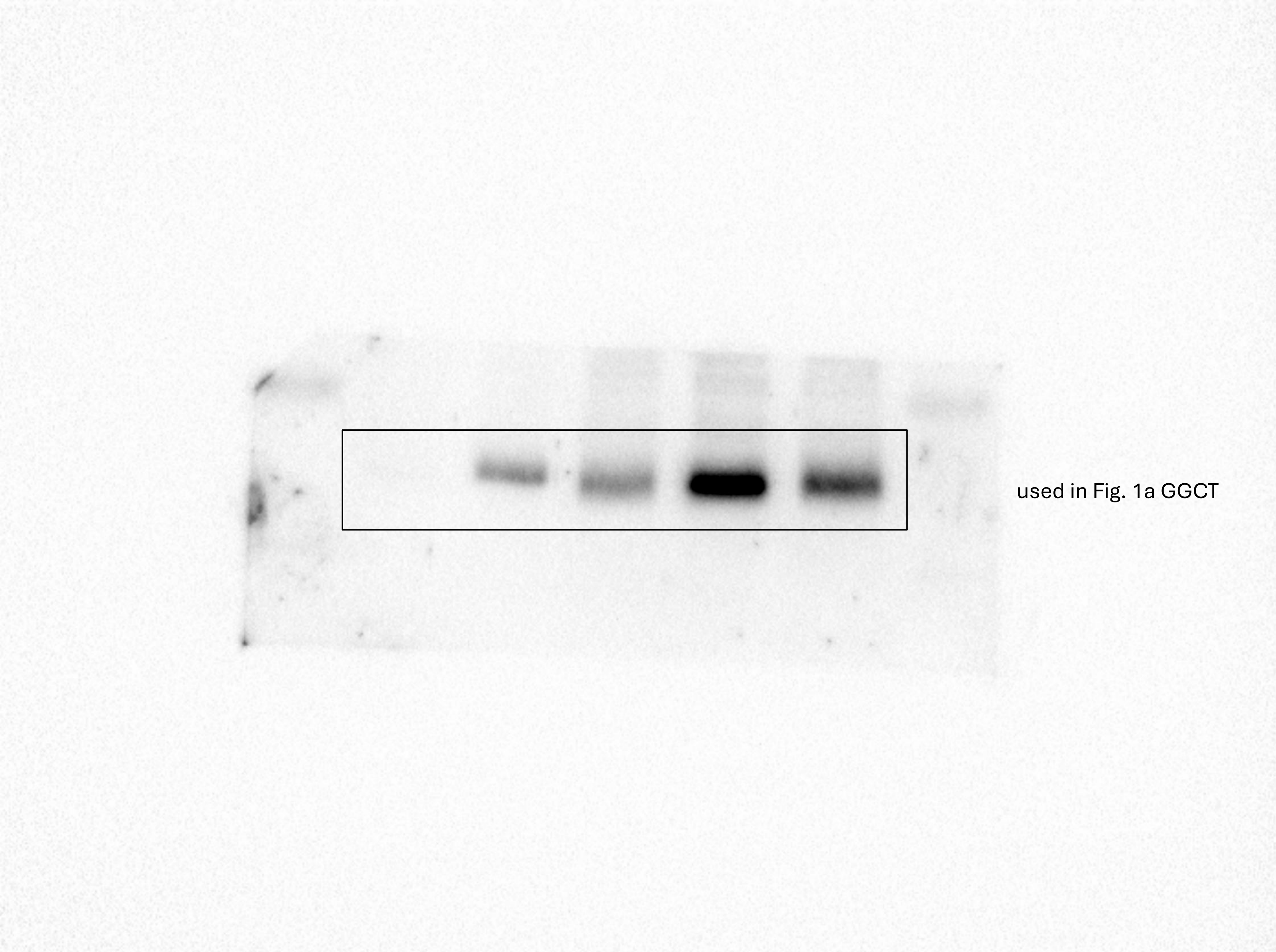

used in Fig. 1a GGCT

Ladder | Astrocyte | U87MG | U251 | A172 | T98 | Ladder

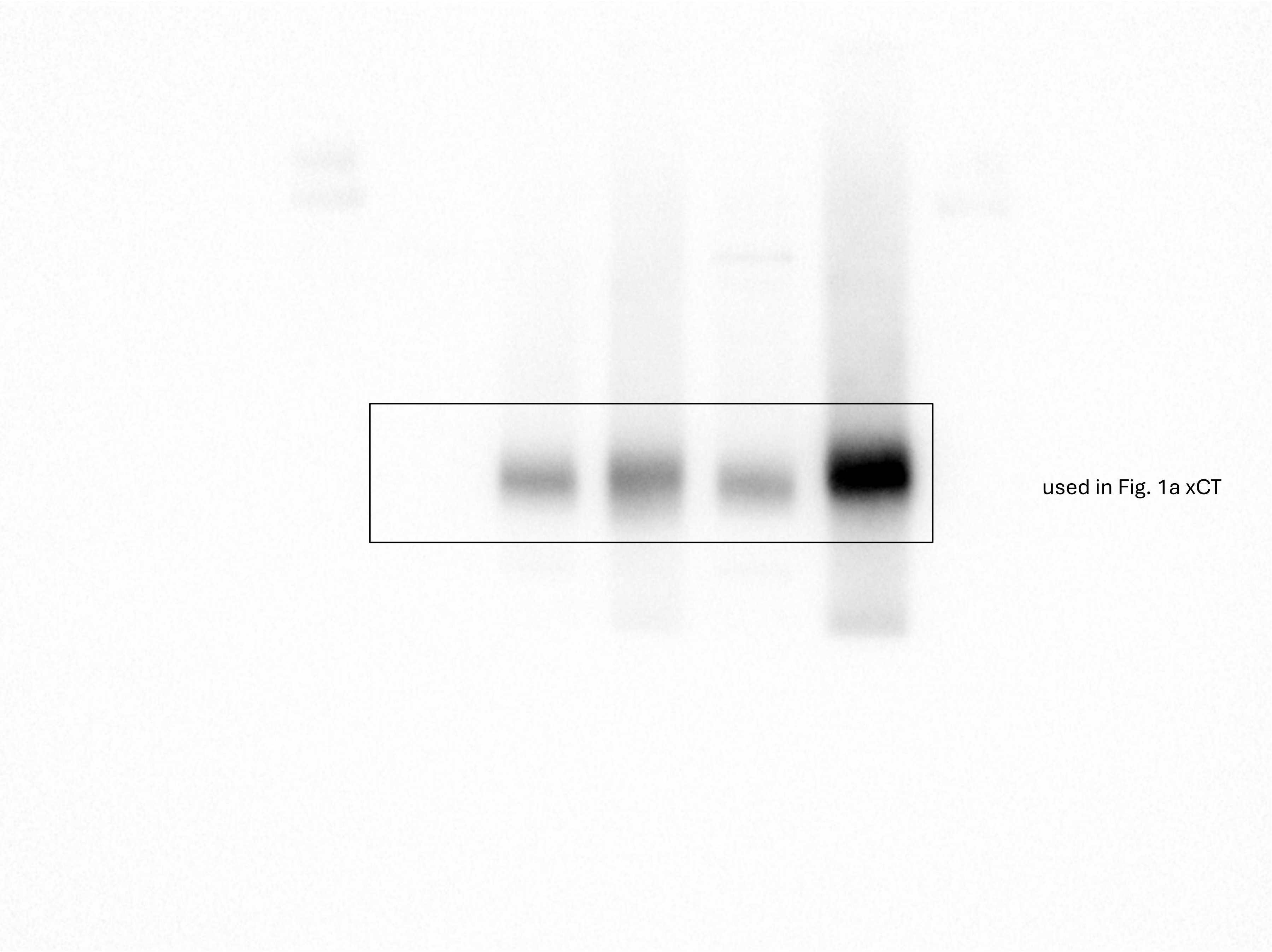

Ladder | Astrocyte | U87MG | U251 | A172 | T98 | Ladder

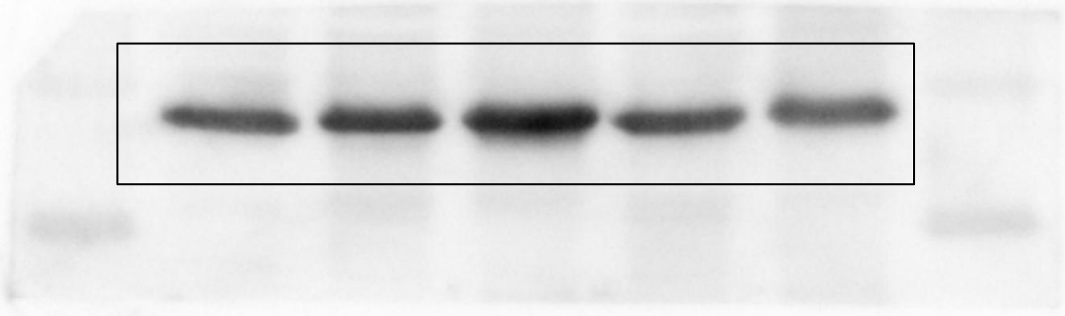

used in Fig. 1a β-actin

Ladder | Astrocyte | U87MG | U251 | A172 | T98 | Ladder

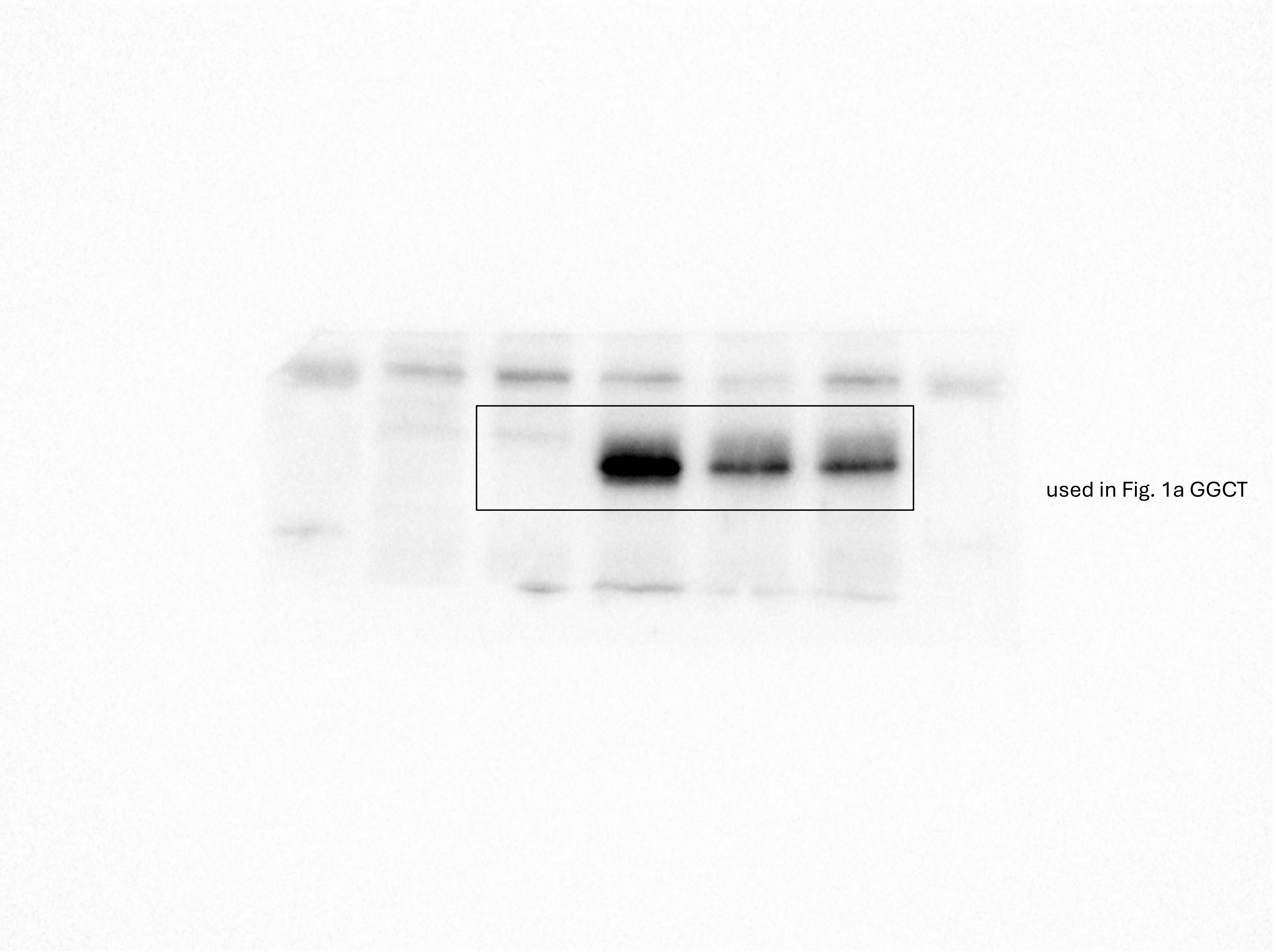
used in Fig. 1a GGCT

Ladder | Astrocyte | NIH-3T3 | GSC1 | GSC2 | GSC3 | Ladder

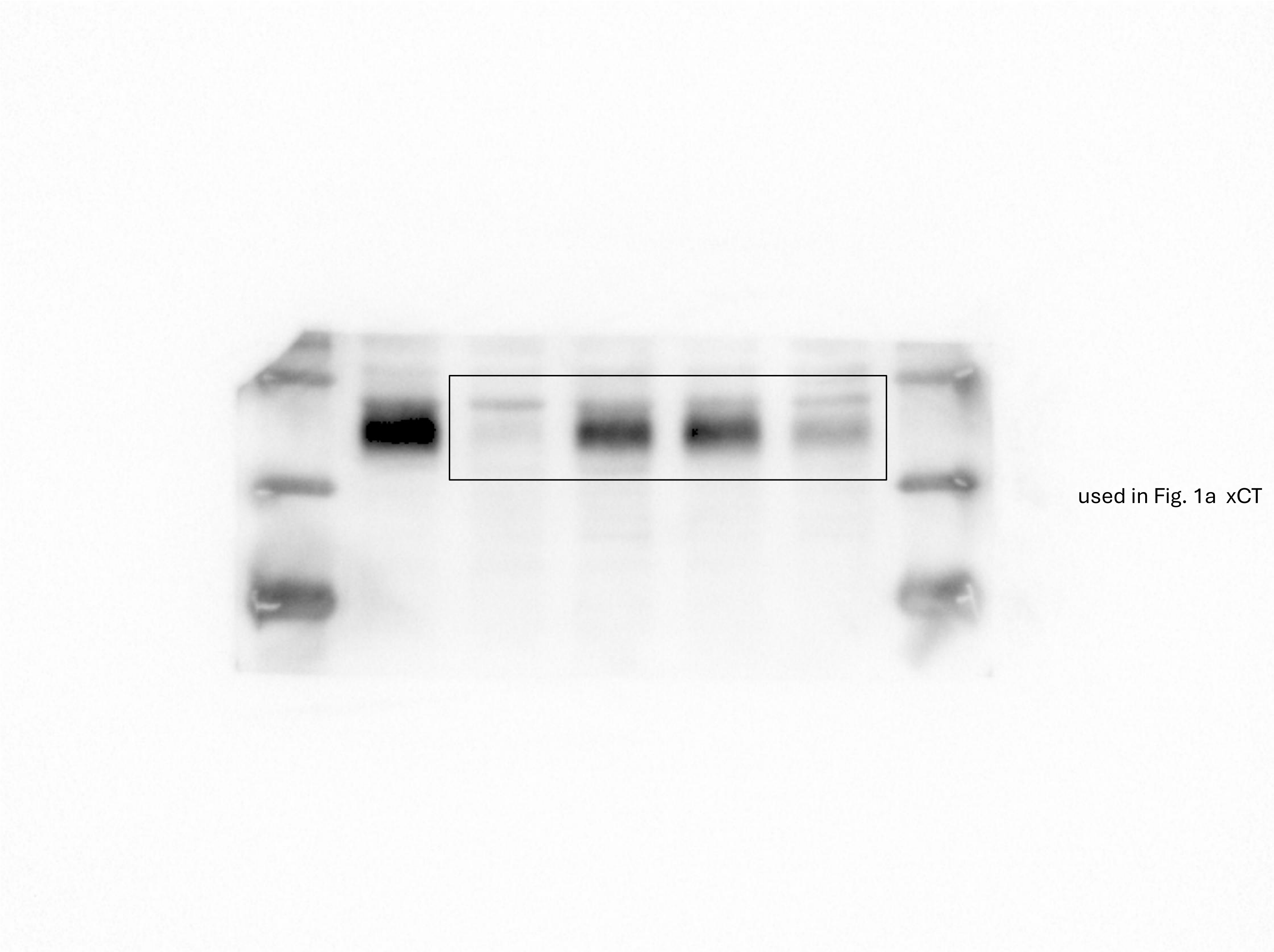

Ladder | Astrocyte | NIH-3T3 | GSC1 | GSC2 | GSC3 | Ladder

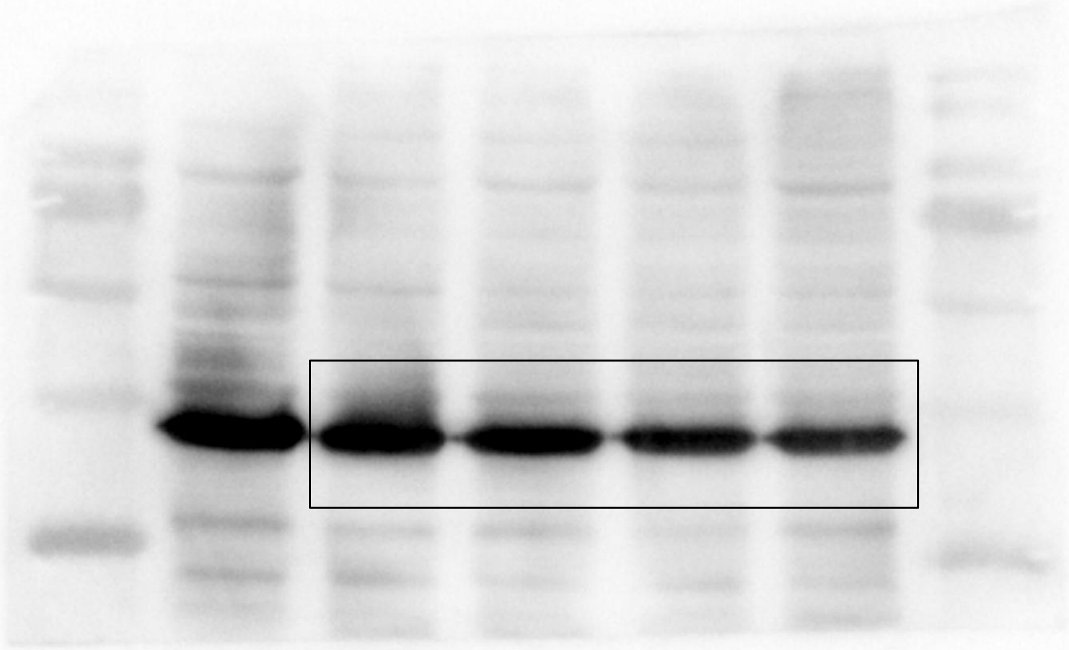

used in Fig. 1a β-actin

Ladder | Astrocyte | NIH-3T3 | GSC1 | GSC2 | GSC3 | Ladder

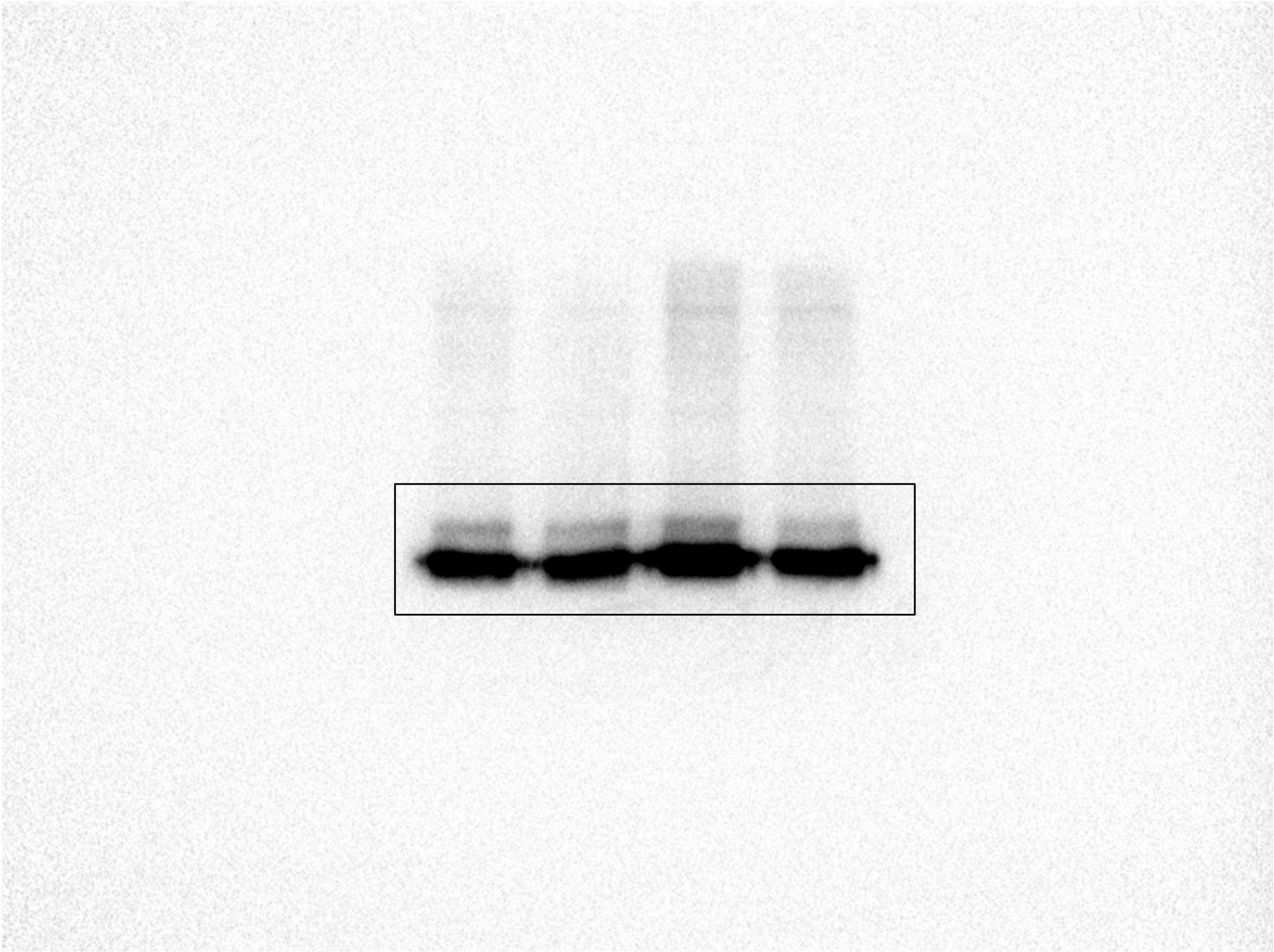

used in Supplemental
Fig. 2a ACSL4

Ladder | U87MG_DMSO | U87MG_pro-GA 60 mM | U87MG_Erastin 0.3 mM | U87MG_combi | Ladder

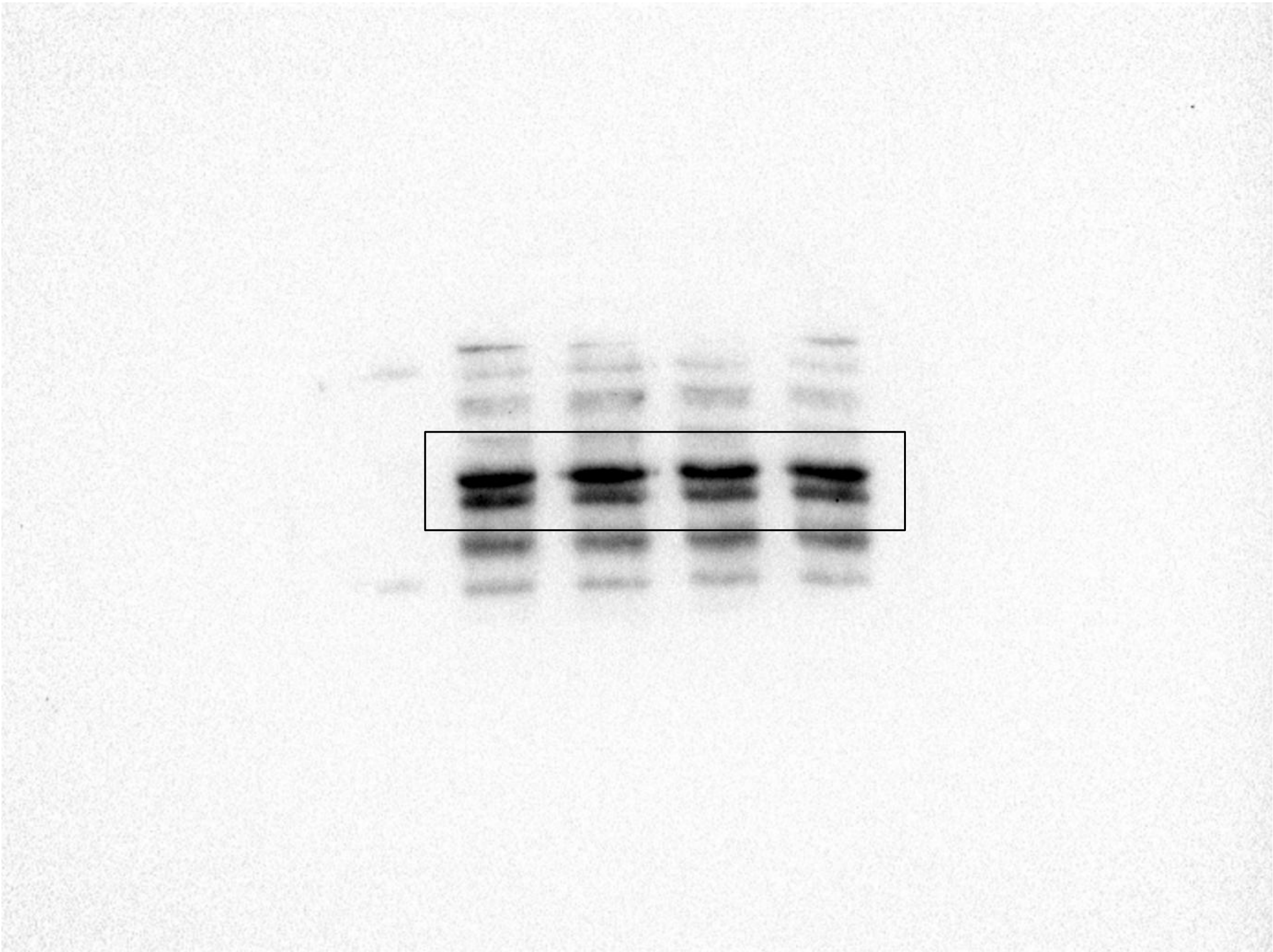

used in in Supplemental
Fig. 2a LPCAT3

Ladder | U87MG_DMSO | U87MG_pro-GA 60 mM | U87MG_Erastin 0.3 mM | U87MG_combi | Ladder

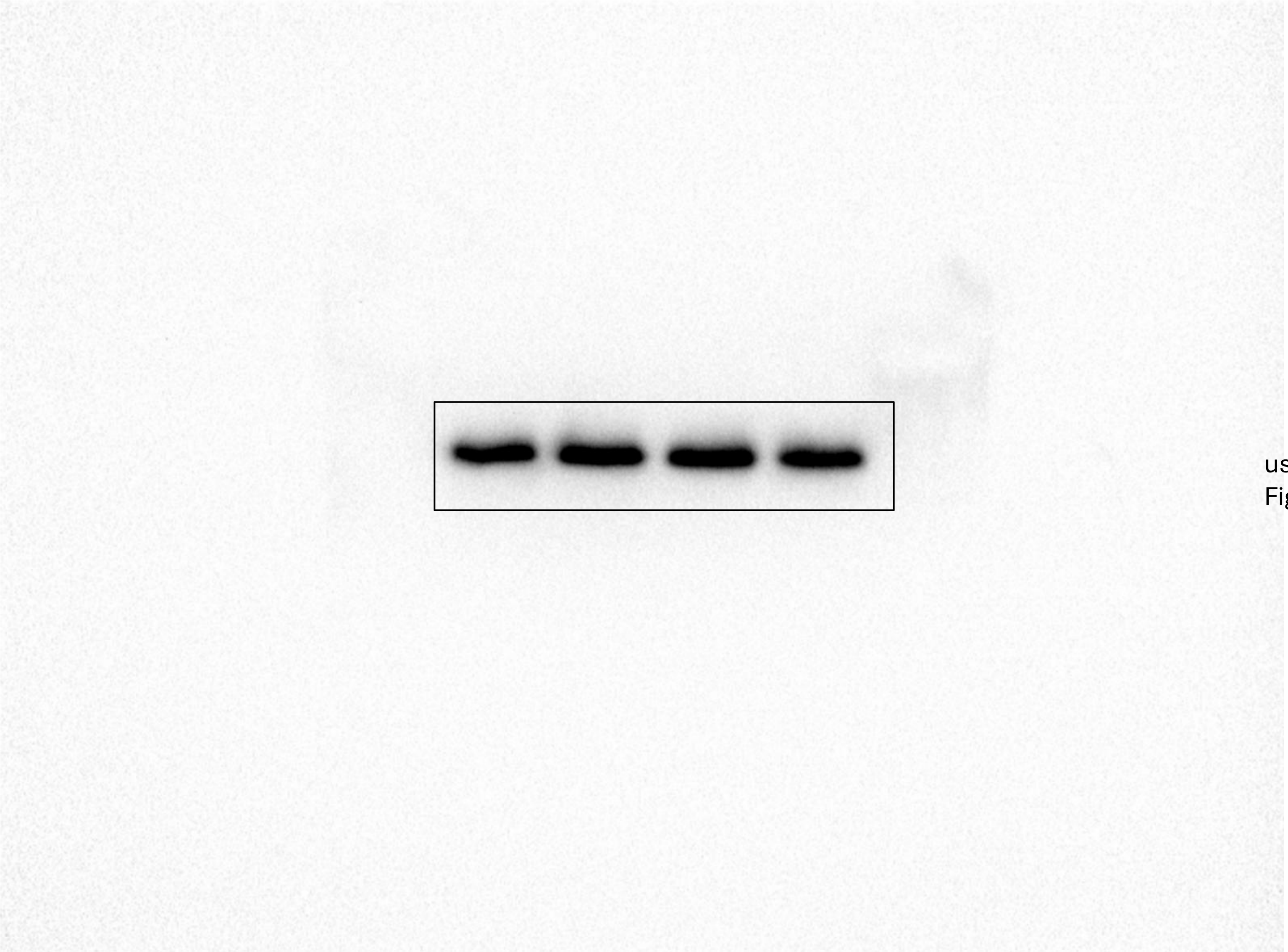

used in in Supplemental
Fig. 2a GPX4

Ladder | U87MG_DMSO | U87MG_pro-GA 60 mM | U87MG_Erastin 0.3 mM | U87MG_combi | Ladder

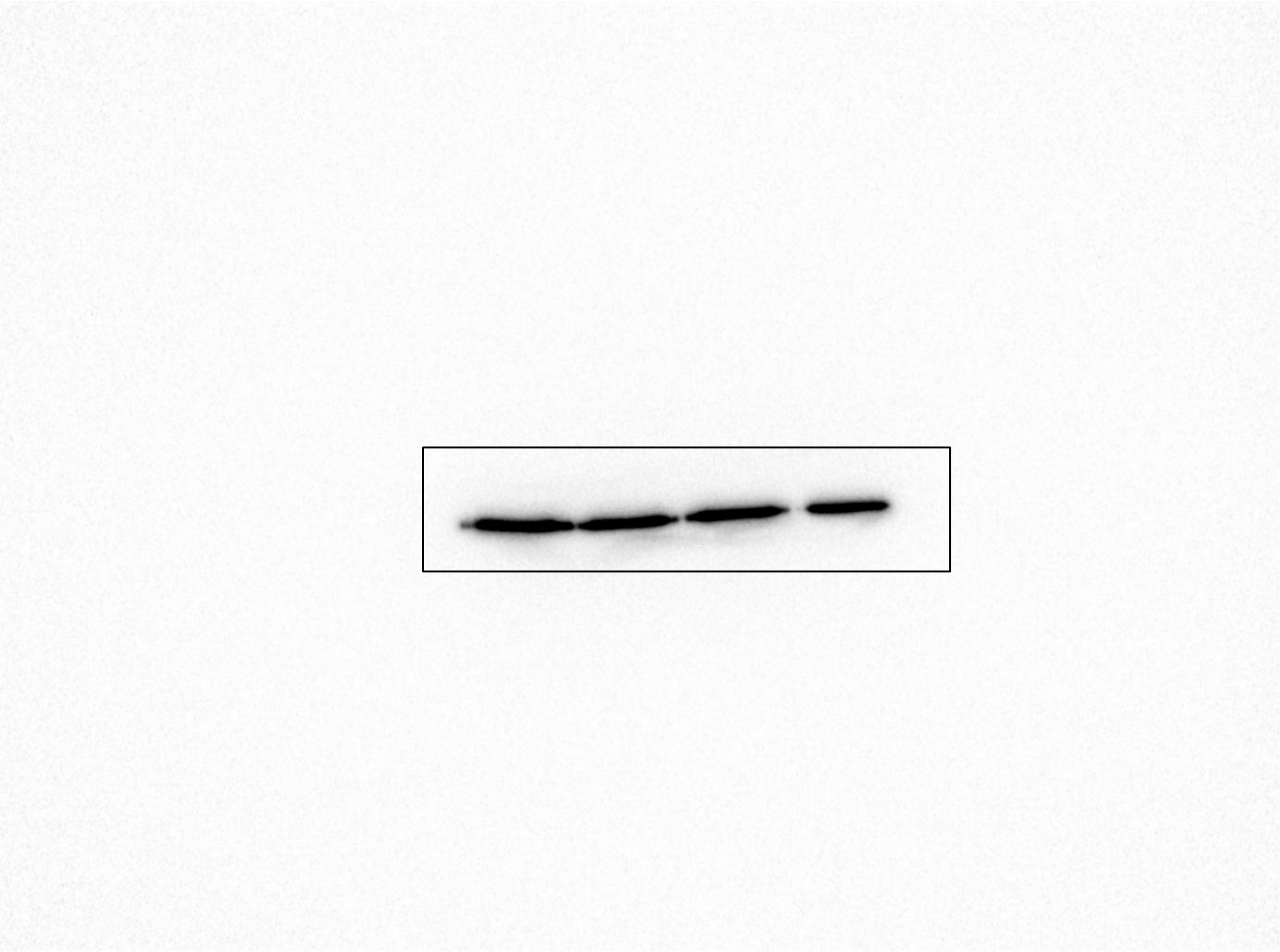

used in in Supplemental
Fig. 2a β-actin

Ladder | U87MG_DMSO | U87MG_pro-GA 60 mM | U87MG_Erastin 0.3 mM | U87MG_combi | Ladder

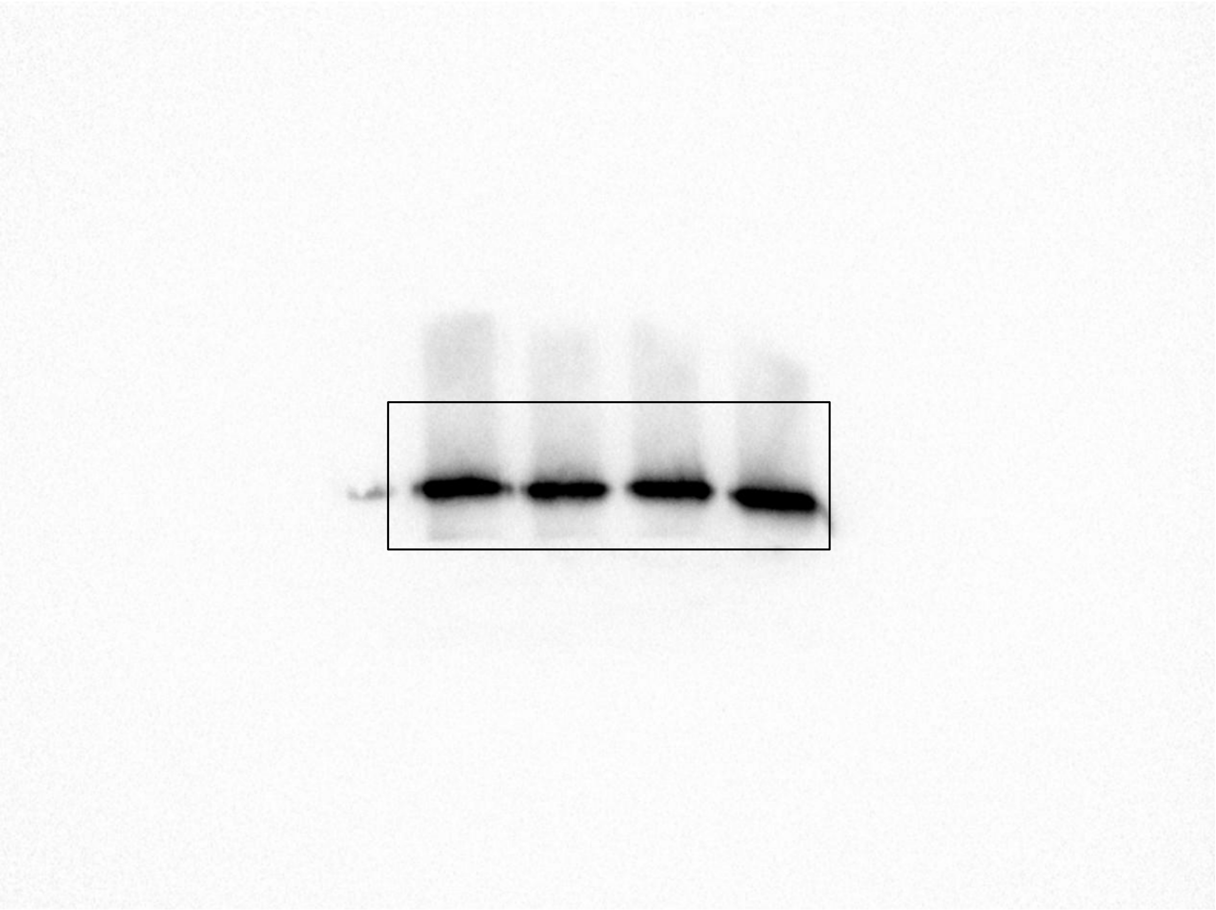

used in Supplemental
Fig. 2a ACSL4

Ladder | GSC_DMSO | GSC_pro-GA 120 mM | GSC_Erastin 0.3 mM | GSC_combi | Ladder

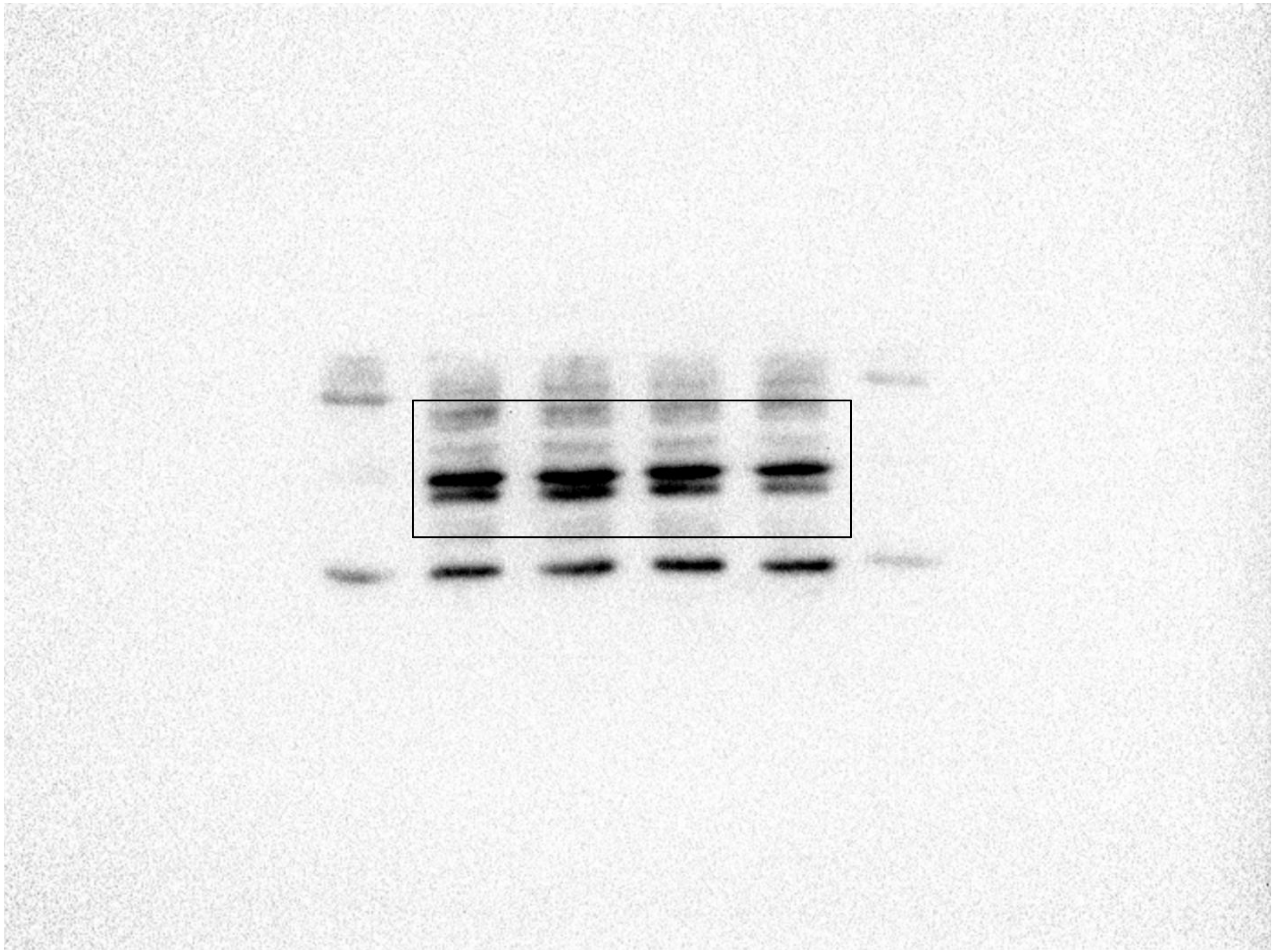

used in Supplemental
Fig. 2a LPCAT3

Ladder | GSC_DMSO | GSC_pro-GA 120 mM | GSC_Erastin 0.3 mM | GSC_combi | Ladder

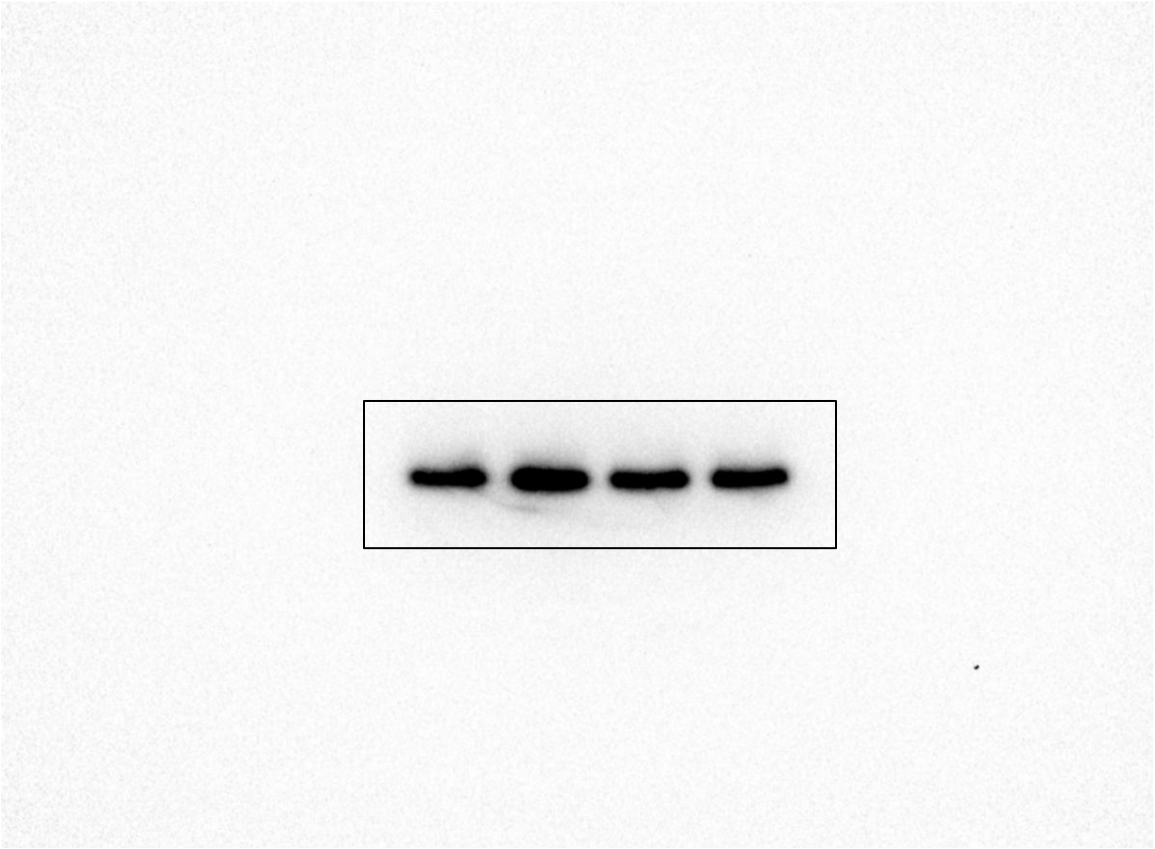

used in Supplemental
Fig. 2a GPX4

Ladder | GSC_DMSO | GSC_pro-GA 120 mM | GSC_Erastin 0.3 mM | GSC_combi | Ladder

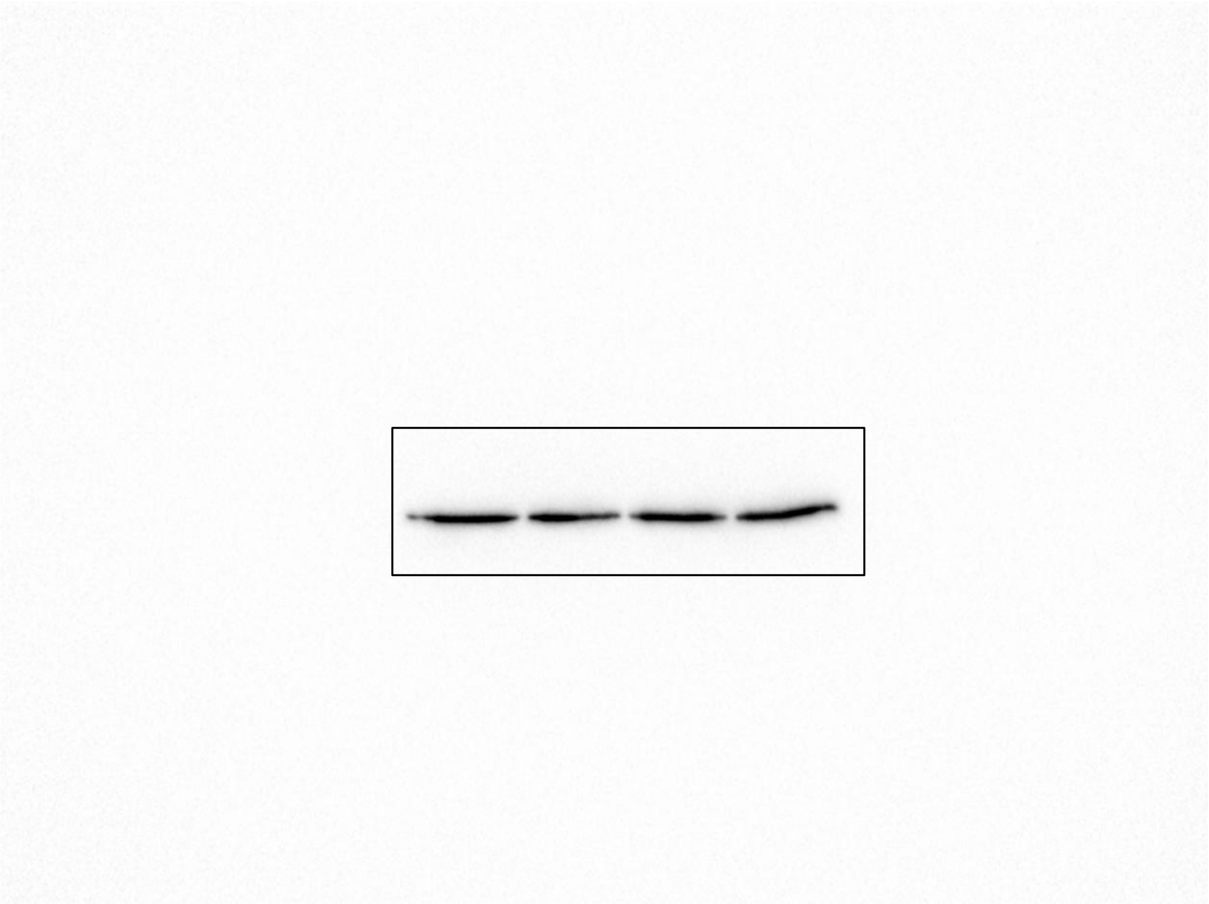

used in Supplemental
Fig. 2a β-actin

Ladder | GSC_DMSO | GSC_pro-GA 120 mM | GSC_Erastin 0.3 mM | GSC_combi | Ladder
